# Supplementary material for: The potential of X-ray computed tomography for xylological and dendrochronological analyses of Egyptian mummy labels
Source: PLoS One. 2024 Jun 28;19(6):e0303695. doi: 10.1371/journal.pone.0303695 (PMC11213358; doi:10.1371/journal.pone.0303695)

PLOS ONE

The potential of X-ray computed tomography for a xylological and dendrochronological analysis of Egyptian mummy labels

--Manuscript Draft--

**Manuscript Number:**	PONE-D-23-11489
**Article Type:**	Research Article
**Full Title:**	The potential of X-ray computed tomography for a xylological and dendrochronological analysis of Egyptian mummy labels
**Short Title:**	The potential of X-ray computed tomography of Egyptian mummy labels
**Corresponding Author:**	François Blondel<br>University of Geneva: Universite de Geneve<br>Geneva, canton of Geneva SWITZERLAND
**Keywords:**	Mummy label<br>Roman Egypt<br>Anatomical identification<br>Dendrochronological analysis<br>Dendrological reading<br>Internal view of wood<br>X-ray tomography imaging
**Abstract:**	X-ray computed tomography (XRCT) imaging allows non-destructive visualization of the structure of various materials. Applied to wooden objects, it allows determination of their morphologies or manufacturing techniques, but also measurement of the growth ring widths. We have tested this approach on a selection of mummy labels. This funerary furniture, made up of endemic or imported tree species, is available in very large quantities in Roman Egypt and thus offers a unique opportunity to build floating or absolutely dated dendrochronological references for this period. We discuss here the contributions and limitations of XRCT for the analysis of these artifacts and show that the tomographic approach allows identification of discriminating markers for the identification of certain species on the transverse plane, but that the insufficient resolution of the tangential and radial planes normally prevents formal identification of species. For the tracery analysis, tomography undeniably helps to improve observations regard concerning the frequency and depth of tool blows, and to highlight structures remaining invisible to the naked eye; XRCT thus provides an unambiguous vision of the cutting methods and calibers used. It thus provides new information on the silvicultural practices and know-how of Egyptian craftsmen. Finally, the measurement of ring widths on XRCT imagery is also more accurate than what can be achieved by traditional dendrochronological approaches, especially in the case of cutting on a slab. The approach also confirms the limited dendrochronological potential of local broadleaved species for dendrochronological approaches due to unreadable or poorly visible tree rings and mostly short ring series.
**Order of Authors:**	François Blondel
	Gisela Bélot
	Christophe Corona, Dr
	Sabine R. Huebner
	Markus Stoffel
**Additional Information:**	
**Question**	**Response**
**Financial Disclosure**<br><br>Enter a financial disclosure statement that describes the sources of funding for the work included in this submission. Review	Yes.<br>This article is part of a post-doctoral project funded by the SNSF, project n°192176 "The Roman Egypt Laboratory: Climat Change, Societal Transformations, and the Transition to Late Antiquity"<br>web page project: https://ancientclimate.philhist.unibas.ch/en/project/

Powered by Editorial Manager® and ProduXion Manager® from Aries Systems Corporation

the submission guidelines for detailed requirements. View published research articles from *PLOS ONE* for specific examples.<br><br>This statement is required for submission and **will appear in the published article** if the submission is accepted. Please make sure it is accurate.<br><br>**Unfunded studies**<br>Enter: *The author(s) received no specific funding for this work.*<br><br>**Funded studies**<br>Enter a statement with the following details:<br>• Initials of the authors who received each award<br>• Grant numbers awarded to each author<br>• The full name of each funder<br>• URL of each funder website<br>• Did the sponsors or funders play any role in the study design, data collection and analysis, decision to publish, or preparation of the manuscript?<br>• **NO** - Include this sentence at the end of your statement: *The funders had no role in study design, data collection and analysis, decision to publish, or preparation of the manuscript.*<br>• **YES** - Specify the role(s) played.<br><br>* typeset	Web page SNSF :<br>https://www.snf.ch/fr
**Competing Interests**<br><br>Use the instructions below to enter a competing interest statement for this submission. On behalf of all authors, disclose any competing interests that could be perceived to bias this work—acknowledging all financial support and any other relevant financial or non-financial competing interests.<br><br>This statement is required for submission and **will appear in the published article** if the submission is accepted. Please make sure it is accurate and that any funding sources listed in your Funding Information later in the submission form are also	NO authors have competing interests

Powered by Editorial Manager® and ProduXion Manager® from Aries Systems Corporation

declared in your Financial Disclosure statement.<br><br>View published research articles from *PLOS ONE* for specific examples.<br><br>**NO authors have competing interests**<br><br>Enter: *The authors have declared that no competing interests exist.*<br><br>**Authors with competing interests**<br><br>Enter competing interest details beginning with this statement:<br><br>*I have read the journal's policy and the authors of this manuscript have the following competing interests: [insert competing interests here]*<br><br>* typeset	
**Ethics Statement**<br><br>Enter an ethics statement for this submission. This statement is required if the study involved:<br><br>• Human participants<br>• Human specimens or tissue<br>• Vertebrate animals or cephalopods<br>• Vertebrate embryos or tissues<br>• Field research<br><br>Write "N/A" if the submission does not require an ethics statement.<br><br>General guidance is provided below. Consult the submission guidelines for detailed instructions. **Make sure that all information entered here is included in the Methods section of the manuscript.**	N/A

**Format for specific study types**<br><br>**Human Subject Research (involving human participants and/or tissue)**<br>• Give the name of the institutional review board or ethics committee that approved the study<br>• Include the approval number and/or a statement indicating approval of this research<br>• Indicate the form of consent obtained (written/oral) or the reason that consent was not obtained (e.g. the data were analyzed anonymously)<br><br>**Animal Research (involving vertebrate animals, embryos or tissues)**<br>• Provide the name of the Institutional Animal Care and Use Committee (IACUC) or other relevant ethics board that reviewed the study protocol, and indicate whether they approved this research or granted a formal waiver of ethical approval<br>• Include an approval number if one was obtained<br>• If the study involved *non-human primates*, add *additional details* about animal welfare and steps taken to ameliorate suffering<br>• If anesthesia, euthanasia, or any kind of animal sacrifice is part of the study, include briefly which substances and/or methods were applied<br><br>**Field Research**<br><br>Include the following details if this study involves the collection of plant, animal, or other materials from a natural setting:<br>• Field permit number<br>• Name of the institution or relevant body that granted permission	
**Data Availability**<br><br>Authors are required to make all data underlying the findings described fully available, without restriction, and from the time of publication. PLOS allows rare exceptions to address legal and ethical concerns. See the PLOS Data Policy and FAQ for detailed information.	Yes - all data are fully available without restriction

A Data Availability Statement describing where the data can be found is required at submission. Your answers to this question constitute the Data Availability Statement and **will be published in the article**, if accepted.<br><br>**Important:** Stating 'data available on request from the author' is not sufficient. If your data are only available upon request, select 'No' for the first question and explain your exceptional situation in the text box.<br><br>Do the authors confirm that all data underlying the findings described in their manuscript are fully available without restriction?	
**Describe where the data may be found in full sentences. If you are copying our sample text, replace any instances of XXX with the appropriate details.**<br><br>• If the data are **held or will be held in a public repository**, include URLs, accession numbers or DOIs. If this information will only be available after acceptance, indicate this by ticking the box below. For example: *All XXX files are available from the XXX database (accession number(s) XXX, XXX.).*<br>• If the data are all contained **within the manuscript and/or Supporting Information files**, enter the following: *All relevant data are within the manuscript and its Supporting Information files.*<br>• If neither of these applies but you are able to provide **details of access elsewhere**, with or without limitations, please do so. For example:<br><br>*Data cannot be shared publicly because of [XXX]. Data are available from the XXX Institutional Data Access / Ethics Committee (contact via XXX) for researchers who meet the criteria for access to confidential data.*<br><br>*The data underlying the results presented in the study are available from (include the name of the third party*	All relevant data are within the manuscript and its Supporting Information files.

and contact information or URL).<br>• This text is appropriate if the data are owned by a third party and authors do not have permission to share the data.<br><br>* typeset	
Additional data availability information:	

Powered by Editorial Manager® and ProduXion Manager® from Aries Systems Corporation

Manuscript
Click here to access/download;Manuscript;Blondel et al. 2023.docx

The potential of X-ray computed tomography for a xylological and dendrochronological analysis of Egyptian mummy labels

The potential of X-ray computed tomography of Egyptian mummy labels

François Blondel[1, 2], Gisela Bélot[3], Christophe Corona[1, 4], Sabine R. Huebner[5], Markus Stoffel[1, 6, 7]

[1] Climate Change Impacts and Risks in the Anthropocene (C-CIA) Institute for Environmental Sciences University of Geneva, 66 Bvd Carl Vogt, CH-1205 Geneva, Switzerland.

[2] Laboratory Chrono-environnement, UMR 6249, 16 route de Gray, F-25000 Besançon, France.

[3] Bibliothèque Nationale et Universitaire (BNU), 5 rue Maréchal Joffre, BP 51029, F-67070 Strasbourg, France.

[4] Laboratory of Physical Geography and Environmental, Geolab, UMR 6042, 4 rue Ledru, F-63057 Clermont-Ferrand, Cedex1, France.

[5] Department of Ancient Civilizations, Institute of Ancient History, University of Basel, Petersgraben 51, CH-4051 Basel, Switzerland.

[6] Dendrolab.ch, Department of Earth Sciences, University of Geneva, 13 rue des Maraîchers, CH-1205 Geneva, Switzerland

[7] Department F.-A. Forel for Environmental and Aquatic Sciences, University of Geneva, 66 Bvd Carl Vogt, CH-1205 Geneva, Switzerland

Abstract

X-ray computed tomography (XRCT) imaging allows non-destructive visualization of the structure of various materials. Applied to wooden objects, it allows determination of their morphologies or manufacturing techniques, but also measurement of the growth ring widths. We have tested this approach on a selection of mummy labels. This funerary furniture, made up of endemic or imported tree species, is available in very large quantities in Roman Egypt and thus offers a unique opportunity to build floating or absolutely dated dendrochronological references for this period. We discuss here the contributions and limitations of XRCT for the analysis of these artifacts and show that the tomographic approach allows identification of discriminating markers for the identification of certain species on the transverse plane, but that the insufficient resolution of the tangential and radial planes normally prevents formal identification of species. For the tracery analysis, tomography undeniably helps to improve observations regard concerning the frequency and depth of tool blows, and to highlight structures remaining invisible to the naked eye; XRCT thus provides an unambiguous vision of the cutting methods and calibers used. It thus provides new information on the silvicultural practices and know-how of Egyptian craftsmen. Finally, the measurement of ring widths on XRCT imagery is also more accurate than what can be achieved by traditional dendrochronological approaches, especially in the case of cutting on a slab. The approach also confirms the limited dendrochronological potential of local broadleaved species for dendrochronological approaches due to unreadable or poorly visible tree rings and mostly short ring series.

Introduction

Dendrochronological analyses are sometimes performed on wooden objects from museum collections [1]: these may include architectural wood (e.g., boats, construction wood) [2,3], domestic objects [4,5] or pieces of art [6,7]. Analyses on objects conserved in museums are performed on woods covering a wide period, often ranging from the Neolithic to the Common Era. They necessarily have to rely, however, when possible, on non-invasive approaches [1] such as high-resolution digital photography, hyper-spectral imaging, fast ion beam analysis [8] or X-ray computer tomography (XRCT). The latter allows visualization of the internal structure of wood without affecting its integrity [9,4,10,11,7]. It allows an internal view of the anatomy of the species used, but also provides archaeological (i.e. cutting and collection methods, exploited calibers) and socio-environmental (e.g., silvicultural practices) information [12-14]. At the same time XRCT also allows highlighting the dendrochronological potential of some species [9]. This potential depends in particular on the existence of identifiable boundaries between growth rings, but also on their annual character. Here, XRCT was tested on a batch of mummy labels from Roman Egypt. Mainly dated from the 1st to the 4th centuries, these wooden objects can provide information on the shaping techniques used, the selection of species for funerary purposes and the positions of cuts within the trees that were exploited [15]. Egypt is an auspicious territory to assess organic archives (papyrus, wood, leather, etc.) thanks to its arid environment in which objects have been conserved over millennia. The objective of this article is to perceive the anatomical, dendrochronological and technological potential of XRCT applied to mummy labels from the National and University Library (BNU) of Strasbourg. In addition, and due to the origin of the wood used, documenting the dendrochronological potential of certain species would eventually allow the establishment of several reference chronologies from the Eastern Mediterranean for the Roman period, which currently exist only in the form of floating chronologies [16-19].

Materials and Method

Mummy labels from the BNU in Strasbourg

The BNU of Strasbourg has 256 mummy labels and various documentary wooden labels [20]. The collection was established between 1895 and 1913 by the Egyptologist Wilhelm Spiegelberg, and contains mainly labels from Sohag and Akhmim in Egypt, dating to the Greco-Roman period.

Spiegelberg acquired 206 labels from Robert Forrer (archaeologist, writer, collector) and made further acquisitions of demotic and Greek-Demotic labels – including tax receipts and accounting records from Gebelein (Pathyris) and Akhmim (Panopolis) – and even 8 h[illegible]atic labels. This collection became an important source for Spiegelberg's onomastic study of the *Ägyptische und Griechische Eigennamen* [21]. Many Egyptologists, including F. Preisigke (between 1913 and 1922) or U. Kaplony-Heckel (in 1966) contributed to the regular edition of the texts of the collection, but it was necessary to wait for the publication of S. P. Vleeming to have a real compendium of the edited texts of the Demotic and Greek labels, including the collection at BNU Strasbourg [22].

These labels, most often made of wood, were pierced to be attached to embalmed mummies when the latter were moved from the place of mummification to the place of burial [23] (Fig 1). The great diversity of their shapes, materials (wood, stone, terracotta, enameled glass), the species of wood used and the inscriptions do not always allow identification of the origin of the manufacturers (mummification workshops, families or wood craftsmen) [15] (Blondel *et al.,* 2023). The labels contain more or less information on the embalmed bodies, that is name and origin (filiation), age and profession of the deceased, sometimes a name of a town or region, the mummification method, or a burial destination [23] (Gaudard *et al.,* 2009). The text is in Greek or Demotic, often both. It may be written in black ink (more rarely red), or sometimes engraved [15,20]. These labels served two main functions: signaling to identify the body until burial; and religious and protective functions to accompany the deceased to the afterlife.

Figure 1 : Some examples of mummy labels from the collection of the BNU of Strasbourg.

The labels vary greatly in form and were classified using the typology proposed by Blondel *et al.* [15], which in turn was inspired by Quaegebeur [24], Gaudard *et al*. [23] and Worp [25]. They are sometimes made from wood whose reuse is attested by marks (i.e. remnant of a groove, dowel hole, nail hole) that is unrelated to the purpose of the label [15]. They are most often obtained by sawing, and then, for the most part, flattened to be inscribed or engraved. Some clumsiness in writing attests to the limited expertise within funerary establishments, woodworking shops, or families. Other labels, ~~on the other hand,~~ show a perfect mastery of woodworking, writing, and even drawing (e.g., representations of religious symbols). These labels do not come from the most disadvantaged social strata since they involve an expensive mummification process [26].

X-Ray Computer Tomography

An EasyTom 150/160 X-ray tomograph (RX Solutions), able to scan objects up to 380 mm in height and 180 mm in diameter, was used on a selection of mummy labels from the BNU. Placed on a support that guarantees protection and stability, each piece was placed on a rotating plate located between the X-ray source and the 2D detector (Fig 2) and then X-rayed at 360°. The resolution of the acquisitions varied between 11 and 42 µm with 2016 projections (that is about 20 images on average per projection). Each image was then reconstructed by filtered retroprojection using the XAct software (RX Solutions). The acquired images were then visualized and processed with the VGStudio Max software (VolumeGraphics) to restore a 3D view of the object. The tomograph and software also allows to work in 2D on the transverse, tangential and radial planes to visualize the internal structure of the wood and to highlight its anatomical characteristics without physical cutting.

Figure 2 : Location of a label (HO137) on its support/vice in the tomograph between the X-ray source and the 2D detector and detail of the tomographed area on the label (Coll. and photogr. BNU de Strasbourg).

Selection of tomographed labels

The selection of tomographed mummy labels was based on their anatomical (taxonomic diversity) or dendrochronological (number of rings present to perform dating) potential. Based on these criteria, 38 labels were selected from a body of imported coniferous (n=11), imported broadleaved (n=10) and local (n=17) species. Tomographic acquisitions were taken on a small portion of the label to obtain a complete dendrochronological sequence at very high resolution. The resolution varied according to the width (32-95 mm) and thickness (5-16 mm) of the labels due to the absorption of the X-ray beam. The detailed description of each of the selected labels is provided in Table 1. Dated mainly on the basis of style, inscription, and place of provenance, they cover a period from the 1st to the 4th century CE and originate for the most part (27 out of 38) from the necropolis of Sohag (n=21), Bompae (n=3), and Panopolis (n=3) located in Upper Egypt. A vast majority of the labels (n=27) are rectangular in shape, nine are trapezoidal, one is in the shape of a stele with a handle (or handle) and one is in the shape of a *Tabula Ansata* (see Blondel *et al.*, 2023 [15] for details on mummy label styles and shapes). No label with a shape close to the square has been tomographed.

Museum inventory	First Edition	Period	Provenance	Format	Tree genus	Dimensions (mm)			Resolution	No. of projections
						Length	width	Thickness		
Ho / PH / T. 018	Short Texts 1 496	Unknown	Unknown	Rectangular	local broadleaves ?	14,4	4,1	0,9	30 µm	1293
Ho / PH / T. 028	Short Texts 2 822, SB 1 5441, C.Étiq.Mom. 1730	225 - 275 CE	Sohag	Rectangular	local broadleaves ?	12,1	6,45	0,95	40 µm	1285
Ho / PH / T. 033	SB 1 5398, T.Spiegelberg 82, C.Étiq.Mom. 421	I - IV CE	Sohag	Rectangular	imported conifers	11,5	4,5	0,8	27 µm	1259
Ho / PH / T. 041	Short Texts 2 756, SB 1 5448, C.Étiq.Mom. 1731	225 - 275 CE	Sohag	Rectangular	local broadleaves	13,2	5,95	0,65	36 µm	1206
Ho / PH / T. 042	SB 1 5449, C.Étiq.Mom. 1732	100 - 299 CE	Sohag	Rectangular	imported broadleaves ?	11,85	5,1	0,65	31 µm	1265
Ho / PH / T. 043	Short Texts 2 703, SB 1 5450, C.Étiq.Mom. 1733	200 - 225 CE	Sohag	Rectangular	imported conifers	13,2	5,6	0,8	35 µm	1264
Ho / PH / T. 047	SB 1 5396, T.Spiegelberg 80, C.Étiq.Mom. 419	I - IV CE	Sohag	Rectangular	local broadleaves	11,9	5,9	1,05	35 µm	1279
Ho / PH / T. 058	SB 1 5372, T. Spiegelberg, 55	Unknown	Unknown	Rectangular	local broadleaves	12,4	5,35	0,8	32 µm	1279
Ho / PH / T. 061	SB 1 5455, C.Étiq.Mom. 459, T.Spiegelberg 95	Unknown	Unknown	Rectangular	local broadleaves ?	12,25	5,4	0,8	32 µm	1302
Ho / PH / T. 065	SB 1 5458, C.Étiq.Mom. 461	Unknown	Unknown	Trapezoidal	imported broadleaves ?	12,2	5,3	1,15	31 µm	1279
Ho / PH / T. 068	SB 1 5389, T.Spiegelberg 73, C.Étiq.Mom. 414	I - IV CE	Sohag	Rectangular	imported broadleaves ?	15,5	7,1	0,8	40 µm	1279
Ho / PH / T. 069	SB 1 5393, T.Spiegelberg 77, C.Étiq.Mom. 417	I - IV CE	Sohag	Rectangular	local broadleaves	14,85	5,5	0,8	30 µm	1279
Ho / PH / T. 085	SB 1 5373, T.Spiegelberg 56, C.Étiq.Mom. 401	I - IV CE	Unknown	Rectangular	imported conifers	11,5	5,5	0,8	35 µm	1279
Ho / PH / T. 095	SB 1 5470, C.Étiq.Mom. 1903	100 - 299 CE	Sohag	Rectangular	local broadleaves	14,4	5,65	1,35	34 µm	1279
Ho / PH / T. 097	SB 1 5472, C.Étiq.Mom. 1742	100 - 299 CE	Sohag	Rectangular	local broadleaves	11,2	5	1,05	32 µm	1265
Ho / PH / T. 104	SB 1 4192, T.Spiegelberg 14, C.Étiq.Mom. 1707, Short Texts 2 824	200 - 299 CE	Bompae	Rectangular	local broadleaves	12,2	6,4	0,95	37 µm	1279
Ho / PH / T. 105	SB 1 5397, T.Spiegelberg 81, C.Étiq.Mom. 420	I - IV CE	Sohag	Rectangular	local broadleaves	11,25	4,35	1,25	26 µm	1268
Ho / PH / T. 106	Short texts 1 594	Unknown	Unknown	Rectangular	imported conifers	13,7	4,1	1,15	15 µm	1048
Ho / PH / T. 108	SB 1 5417, T.Spiegelberg 101, C.Étiq.Mom. 436	I - IV CE	Sohag	Trapezoidal	imported conifers	11,95	6,35	1,2	20 µm	960
Ho / PH / T. 111	SB 1 4186, T.Spiegelberg 7, C.Étiq.Mom. 343, Short Texts 2 637	100 - 299 CE	Bompae	Rectangular	local broadleaves ?	16,1	4,8	0,75	30 µm	1279
Ho / PH / T. 112	SB 1 5385, T.Spiegelberg 69, C.Étiq.Mom. 411	I - IV CE	Sohag	Trapezoidal	local broadleaves ?	14,4	6,5	1,05	36 µm	1279
Ho / PH / T. 117	SB 1 5480, SB 1 5410, C.Étiq.Mom. 472, T.Spiegelberg 94	I - IV CE	Sohag	Trapezoidal	local broadleaves ?	10,4	6,45	1	38 µm	1231
Ho / PH / T. 132	SB 1 5487, C.Étiq.Mom 1926	100 - 299 CE	Sohag	Trapezoidal	local broadleaves	14,55	6,5	1,35	38 µm	1263
Ho / PH / T. 137	Short Texts 2 664, SB 1 5489, C.Étiq.Mom. 478	100 - 299 CE	Sohag	Rectangular	imported broadleaves	11,4	4,4	0,5	32 µm	1646
Ho / PH / T. 138	Short Texts 2 794, SB 1 5490, C.Étiq.Mom. 479	200 - 299 CE	Sohag	Trapezoidal	imported broadleaves	10,45	5	0,85	31 µm	1279
Ho / PH / T. 141	SB 1 5492, C.Étiq.Mom. 481	100 - 299 CE	Sohag	Rectangular	imported broadleaves ?	11,3	4,8	0,65	30 µm	1279
Ho / PH / T. 162	SB 1 5502, C.Étiq.Mom. 488	100 - 299 CE	Sohag	Rectangular	local broadleaves	17,7	5,7	1,4	34 µm	1266
Ho / PH / T. 170	Short Texts 2 683, SB 1 5506, C.Étiq.Mom. 1753	200 - 225 CE	Sohag	Trapezoidal	imported conifers	14,7	5,8	1,05	33 µm	1279
Ho / PH / T. 172	SB 1 4194, T.Spiegelberg 17, SB 1 5507, C.Étiq.Mom. 348, C.Étiq.Mom. 490, Short Texts 2 757	220 - 299 CE	Panopolis (?)	Rectangular	imported conifers	13,5	6,9	1,2	42 µm	1279
Ho / PH / T. 182	SB 1 4200, T.Spiegelberg 23, C.Étiq.Mom. 1709, Short Texts 2 684	200 - 250 CE	Bompae	Rectangular	imported conifers	11,9	4,8	0,9	30 µm	1279
Ho / PH / T. 184	SB 1 5511, C.Étiq.Mom. 1754, Short Text 2 750	100 - 299 CE	Panopolis/Bompae (?)	Rectangular	imported conifers	11,9	5,2	1,1	32 µm	1269
Ho / PH / T. 199	T.Spiegelberg 41, Short Texts 2 574	III CE	Panopolis (?)	Trapezoidal	imported broadleaves	8,3	3,2	0,5	11 µm	1079
Ho / PH / T. 210	SB 1 5525, C.Étiq.Mom. 502	Unknown	Unknown	Stela-shape with handle	imported broadleaves	12,55	8	0,85	28 µm	1112
Ho / PH / T. 211	SB 1 5526, C.Étiq.Mom. 1978, T.Spiegelberg 107, SB 1 5423 descr.	I - IV CE	Sohag	Trapezoidal	imported conifers	12,8	6,1	1	35 µm	1310
Ho / PH / T. 225	SB 1 5536, C.Étiq.Mom. 510, Short Texts 2 741	100 - 299 CE	Panopolis (?)	Rectangular	imported conifers	10,3	5	0,85	30 µm	1248
Ho / PH / T. 226	SB 1 5537, C.Étiq.Mom. 2122	Unknown	Unknown	Tabula ansata	local broadleaves	23,55	9,5	1,2	32 µm	1062
Ho / PH / T. 229	SB 1 5538, C.Étiq.Mom. 1936	Roman	Unknown	Rectangular	imported broadleaves	15,75	9,25	1,6	31 µm	1140
Ho / PH / T. 255	Unknown	Unknown	Unknown	Rectangular	local broadleaves ?	11,4	7,7	0,6	26 µm	949

Table 1 : Inventory of mummy labels selected for CT with key descriptive information and regarding CT resolution.

Anatomical identifications

Due to the insufficient resolution of XRCT for the tangential and radial planes [9], analyses were limited to the transverse plane as it allows for optimal visualization of growth rings. In the case of broadleaved species, anatomical markers included pore number, shape, and distribution as well as a distinction between earlywood and latewood, ring boundaries, presence or absence of parenchyma, as well as ray widths. In the case of conifer species, the markers are less numerous and obvious. Only the transition from earlywood to latewood, the presence of resin ducts or traumatic cells could be made.

On the basis of these identifiable anatomical markers, we propose several hypotheses concerning the species used. These hypotheses are then validated by comparison with (1) the different species identified on several types of Roman Egyptian furniture (e.g., mummy labels, mummy portraits, everyday objects; [27-30]; and (2) the microscopic wood anatomy and macroscopic characteristics in various anatomical atlases [31-36].

Size estimation and technological observations

The estimation of the caliber of the processed wood allows highlighting possible selection criteria of a tree for the realization of the analyzed objects. The estimation of the distance to the pith was performed from the convergence of the woody radii and the curvature of the rings from the cross-sectional planes obtained with XRCT [37-39,12] (Fig 3). Because tree growth is not perfectly concentric, the distance to the pith is calculated as an average between possible minimum and maximum values. Tree age could not always be estimated due to poorly visible ring boundaries in some species.

Figure 3 : Methodology applied for the estimation of the size of the trees selected for the shaping of mummy labels from the transverse plane of the CTs of the HO229 label cut on back.

The cutting methods are sometimes visually identifiable on the ends of the objects. However, tool marks or secretions sometimes limit these observations. We used XRCT to extend these observations to all tomographed labels. Finally, the 3D model obtained from the XRCT images was also used to highlight tool marks. These were compared to the visual observations made on the labels to evaluate a possible added value of XRCT images.

Ring width measurements

The ring width of conifer labels could be measured from a traditional approach because of the distinct ring boundaries on their faces. Only those with more than 30 rings were measured. Measurements (1/100th mm) were made using (1) a Lintab 5 measuring table equipped with a binocular magnifier and Tsap Win v. 4.82b2 software [40] or (2) from high-resolution photographs (300 dpi minimum) using CooRecorder software [41]. To mitigate errors related to illegible, partial rings or the existence of false rings [42], measurements were made on the flat of both sides of each object and along several transects (Fig 4). The series were synchronized using Tsap Win software. These ring series were compared to the series made on the transverse plane of the CTs. The latter were made and synchronized in the same way, on at least three transects in the acquisition volume, using the CooRecorder and TSAPWIN software.

Figure 4 : Presentation of the two methods of measuring ring widths on label HO184: one directly on the object and the other from the CT, as well as the comparison of the ring widths of the two synchronizations obtained (coll. and photogr. BNU de Strasbourg HO184).

Results

Identification of the species used

Using the anatomical criteria visible in the transverse plane of the XRCT images, we were able to differentiate between imported broadleaved species, those endemic to Egypt and imported conifer species. We were also able to hypothesize which species were used in 27 of the 38 labels for which tomographic analyses were performed (Fig 5). Although the anatomy of labels HO18 and 255 appears to be similar, we could not identify the species in these cases.

Figure 5 : Several tomographic images of the transverse plane of six mummy labels of different species of both local and imported woods, as well as a zoom on their anatomy highlighting the limits of the CT resolutions and their definitive anatomical attribution.

For the imported broadleaved species, we hypothesize that labels HO137, 199, 210 and 229 are made from beech (*Fagus* sp.). This hypothesis is linked to the presence of diffuse pores, a semi-porous zone, and the presence of united to multi-serial woody rays that thicken at the edge of the rings, with the latter being perfectly legible. Similarly, using the criteria described in Table 2, labels HO42 and 141 appear to have been sawn from willow (*Salix* sp.) wood while label HO138 is potentially made from elm (*Ulmus* sp.) wood.

In the case of the endemic broadleaved species, seven labels (HO28, 41, 69, 95, 104, 132, and 226) show (1) groups of two to three diffuse pores, sometimes arranged radially, (2) an initial wood with a semi-porous zone, and (3) broad woody rays, sometimes widening at the ring boundaries. Based on these characteristics, they were probably made of tamarisk (*Tamarix* sp.) wood. From other discriminating criteria (Table 2), labels HO47, 58, and 97 are likely made of fig (*Ficus sycomores*) wood, whereas labels HO111 and 162 are likely to be acacia (*Acacia* sp.), although distinguishing between the two species is sometimes complex from the transverse plane alone. Last least, label HO105 seems to be made of jujube (*Ziziphus spina-christi*) wood.

Imported hardwood					
**Museum inv. num.**	**Pore arrangement**	**Rays width**	**Limit of tree-rings**	**Other anatomical markers**	**Hypothesis species**
Ho / PH / T. 42	Diffuse pores, radially bonded with a slightly semi-porous area	Uniseriate	Distinct	Groups of 2 to 3 pores radially	*Salix sp.*
Ho / PH / T. 65	Diffuse pores (small pores), sometimes with a slightly porous area in the initial wood	Uniseriate ?	More or less distinct		Undetermined
Ho / PH / T. 68	Diffuse pores (small pores), sometimes with a slightly porous area in the initial wood	Uniseriate ?	More or less distinct		Undetermined
Ho / PH / T. 112	Diffuse pores sometimes clustered together or radially, with a semi-porous zone	Uniseriate or 2 seriate ?	More or less distinct		Undetermined
Ho / PH / T. 137	Diffuse pores with a semi-porous zone	Uni- and multiseriate	Distinct	Wider woody rays at the boundary of the ring	*Fagus sp.*
Ho / PH / T. 138	Porous zone composed of a single series of pores	Wide rays	Distinct	Several bands of tangential pores, sometimes slightly oblique with parenchyma	*Ulmus sp. Minor*
Ho / PH / T. 141	Diffuse pores with a semi-porous zone	Uniseriate ?	More or less distinct		*Cf. salix*
Ho / PH / T. 199	Diffuse pores with a semi-porous zone	Uni- and multiseriate	Distinct	Wider woody rays at the boundary of the ring	*Fagus sp.*
Ho / PH / T. 210	Diffuse pores with a semi-porous zone	Uni- and multiseriate	Distinct	Wider woody rays at the boundary of the ring	*Fagus sp.*
Ho / PH / T. 229	Diffuse pores with a semi-porous zone	Uni- and multiseriate	Distinct	Wider woody rays at the boundary of the ring	*Fagus sp.*
**Local hardwood**					
Ho / PH / T. 18	Diffuse pores, grouped by two or three	2 seriate or more	More or less distinct	Numerous xylophagous galleries	Undetermined
Ho / PH / T. 28	Diffuse pores, grouped from two to three pores and/or radially, sometimes with a semi-porous zone	Wide to very wide	More or less distinct	Expansion of the radii at the boundary of the ring	*Tamarix sp.*
Ho / PH / T. 41	Diffuse pores, grouped from two to three pores and/or radially, sometimes with a semi-porous zone	Wide to very wide	More or less distinct	Expansion of the radii at the boundary of the ring	*Tamarix sp.*
Ho / PH / T. 47	Diffuse pores, with group of pores by two or three sometimes arranged radially	Wide rays	unreadable	Alternating parenchyma bands and fiber	*Ficus sp.*
Ho / PH / T. 58	Diffuse pores, with group of pores by two or three sometimes arranged radially	Wide rays	unreadable	Alternating parenchyma bands and fiber	*Ficus sp.*
Ho / PH / T. 61	Diffuse pores (small pores) not very close together	Uniseriate ?	unreadable		Undetermined
Ho / PH / T. 69	Diffuse pores, grouped from two to three pores and/or radially, sometimes with a semi-porous zone	Wide to very wide	More or less distinct	Expansion of the radii at the boundary of the ring	*Tamarix sp.*
Ho / PH / T. 95	Diffuse pores, grouped from two to three pores and/or radially, sometimes with a semi-porous zone	Wide to very wide	More or less distinct	Expansion of the radii at the boundary of the ring	*Tamarix sp.*
Ho / PH / T. 97	Diffuse pores, with group of pores by two or three sometimes arranged radially	Wide rays	unreadable	Alternating parenchyma bands and fiber	*Ficus sp.*
Ho / PH / T. 104	Diffuse pores, grouped from two to three pores and/or radially, sometimes with a semi-porous zone	Wide to very wide	More or less distinct	Expansion of the radii at the boundary of the ring	*Tamarix sp.*
Ho / PH / T. 105	Diffuse pores, with group of pores by two to four arranged radially, rarely isolated	Uniseriate ?	More or less distinct	Thin band of parenchyma	*Ziziphus sp.*
Ho / PH / T. 111	Diffuse pores, with groups of pores by two or in clusters and sometimes presenting a semi-porous zone	Wide rays	unreadable	Alternating parenchyma bands and fiber	*Cf. Ficus ou acacia*
Ho / PH / T. 117	Diffuse pores	Uniseriate	unreadable		Undetermined
Ho / PH / T. 132	Diffuse pores, grouped from two to three pores and/or radially, sometimes with a semi-porous zone	Wide to very wide	More or less distinct	Expansion of the radii at the boundary of the ring	*Tamarix sp.*
Ho / PH / T. 162	Diffuse pores, with groups of pores by two or in clusters and sometimes presenting a semi-porous zone	Wide rays	More or less distinct	Alternating parenchyma bands and fiber	*Cf. Ficus ou acacia*
Ho / PH / T. 226	Diffuse pores, grouped from two to three pores and/or radially, sometimes with a semi-porous zone	Wide to very wide	More or less distinct	Expansion of the radii at the boundary of the ring	*Tamarix sp.*
Ho / PH / T. 255	Diffuse pores with a semi-porous zone in the initial wood	Uniseriate or 2 seriate ?	More or less distinct	Numerous xylophagous galleries	Undetermined

Imported conifers					
**Museum inv. num.**	**Presence of resin canals**	**Limit of tree-rings**	**Transition from initial to final wood**	**Particularity**	**Hypothesis species**
Ho / PH / T. 33	Yes	Distinct	Abrupt transition from initial to final wood	Resin canals predominantly in the final wood and thin final wood	Cf. *Pinus*
Ho / PH / T. 43	No	Distinct	Abrupt transition from initial to final wood	Thin final wood, sometimes tangential parenchyma	Cf. *Cupressus ou Cedrus*
Ho / PH / T. 85	No	Distinct	Gradual transition from initial to final wood	Traumatic canals and tree-rings more or less thin	*Cedrus* sp.
Ho / PH / T. 106	No	Distinct	Abrupt transition from initial to final wood	Very thin tree-rings, thin woody rays (3-5 cells)	*Juniperus* sp.
Ho / PH / T. 108	No	Not always distinct	Gradual transition from initial to final wood	Traumatic canals	Undetermined
Ho / PH / T. 170	Yes	Distinct	Abrupt transition from initial to final wood	Resin canals predominantly in the final wood and thin final wood	Cf. *Pinus*
Ho / PH / T. 172	No	Distinct	Gradual transition from initial to final wood	Traumatic canals	*Cedrus* sp.
Ho / PH / T. 182	Yes	Distinct	More or less abrupt transition between the initial and final wood	Resin canals predominantly in the final wood	Cf. *Pinus*
Ho / PH / T. 184	Yes	Distinct	More or less abrupt transition between the initial and final wood	Resin canals predominantly in the final wood	Cf. *Pinus*
Ho / PH / T. 211	Yes	Distinct	Abrupt transition from initial to final wood	Resin canals predominantly in the final wood	Cf. *Pinus*
Ho / PH / T. 225	No	Distinct	Gradual transition from initial to final wood	Fast growing, very thin final wood	Undetermined

Table 2 : Detailed description of the different anatomical markers observed from the transverse plane of the CTs and to a lesser extent from observations made on the mummy labels.

For conifer species, discriminating markers are poorly present on the transversal plane, rendering species determination complex (Table 2). Five labels (HO33, 170, 182, 184 and 211) are potentially made of pine (*Pinus* sp.) because of the presence of resin ducts mainly observed in the latewood and a more or less abrupt transition from early- to latewood. Two labels (HO85 and 172) without resin ducts in their anatomy, but with traumatic tissue and a gradual transition from earlywood to latewood are probably cedar (*Cedrus* sp.). Label HO43, whose anatomical criteria are similar to cedar, but which shows false rings and an abrupt transition between earlywood and a very thin latewood, is probably made of cypress (*Cupressus sempervirens*). Finally, HO106 on which the resin ducts and traumatic ducts are absent, the transition from earlywood to latewood is abrupt and the growth very slow, was probably from a juniper (*Juniperus* sp.).

Tool marks identified in XRCT

Tool marks, identified by positioning the labels at varying angles and different light orientations, were compared to those detected in the XRCT images. In general, the XRCT images validate the visual observations, but allow a finer interpretation on the orientation of the tool and its degree of penetration into the wood.

Several types of traces were regularly observed: sawing traces and those left by sharp tools (i.e. axe, adze, plane and chisel or simply plane) (Fig 6, Table 3). The sawing marks, whose pitch is identifiable on the XRCT, are visible on the rough faces, mostly without inscriptions. However, some labels are inscribed on the roughly sawn face (e.g. HO106, Fig 6). A total of 21 labels shows traces of cutting tools, but the precise identification of the tool used is complex. However, given the small size of many of the labels, plane-type tools seem to be much more suitable for flattening the face or faces intended to receive the inscription (Fig 6). Three other labels show both types of marks (sawing and cutting tools). Eleven labels do not show any preserved tool marks at all, probably because of wear or erosion of their surfaces or simply because of a completed finish.

Figure 6 : Various tool marks and features recognized on the mummy labels and reinforced, and sometimes enriched, from the CTs.

Museum inventory number	Genus	Hypothesis species	Anatomical morphology	Cutting method	Tool marks	Type of inscription	Estimated diameter (in mm)
Ho / PH / T. 18	Local hardwood ?	Undetermined	Cambium	On a slab	cutting tools and saws	in ink	48
Ho / PH / T. 28	Local hardwood ?	*Tamarix sp.*		On a slab	cutting tool	in ink	186
Ho / PH / T. 33	Imported conifers	Cf. *Pinus*		On a slab	cutting tool	in ink	suparallel
Ho / PH / T. 41	Local hardwood	*Tamarix sp.*		On a slab	saw	in ink	140
Ho / PH / T. 42	Imported hardwood	*Salix sp.*		On a slab	/	in ink	70
Ho / PH / T. 43	Imported conifers	*Cf. Cupressus ou Cedrus*		On mesh	/	in ink	200
Ho / PH / T. 47	Local hardwood	*Ficus sp.*		On a slab	/	in ink	314
Ho / PH / T. 58	Local hardwood	*Ficus sp.*		On a slab and a mesh	/	in ink	suparallel
Ho / PH / T. 61	Local hardwood ?	Undetermined	Bark	On a slab	cutting tool	in ink	83
Ho / PH / T. 65	Imported hardwood ?	Undetermined	Nearby pith	On a slab	cutting tool	in ink	80
Ho / PH / T. 68	Imported hardwood ?	Undetermined		On a slab	cutting tool	in ink	118
Ho / PH / T. 69	Local hardwood	*Tamarix sp.*		On a slab	saw	in ink	228
Ho / PH / T. 85	Imported conifers	*Cedrus* sp.		On mesh	cutting tool	in ink	278
Ho / PH / T. 95	Local hardwood	*Tamarix sp.*		On mesh	cutting tool	in ink	suparallel
Ho / PH / T. 97	Local hardwood	*Ficus sp.*		On mesh	/	in ink	130
Ho / PH / T. 104	Local hardwood	*Tamarix sp.*		On a slab	saw	in ink	178
Ho / PH / T. 105	Local hardwood	*Ziziphus sp.*	Pith	On a slab	cutting tool	in ink	44
Ho / PH / T. 106	Imported conifers	*Juniperus* sp.		On mesh	cutting tools and saws	in ink	suparallel
Ho / PH / T. 108	Imported conifers	Undetermined	Pith	On a slab	cutting tool	in ink	94
Ho / PH / T. 111	Local hardwood ?	*Cf. Ficus ou acacia*	Pith	On a slab	cutting tool	in ink	64
Ho / PH / T. 112	Local hardwood ?	Undetermined		On a slab	cutting tools and saws	in ink	82
Ho / PH / T. 117	Local hardwood ?	Undetermined		On a slab	cutting tool	in ink	104
Ho / PH / T. 132	Local hardwood	*Tamarix sp.*		On a slab and a mesh	/	in ink	134
Ho / PH / T. 137	Imported hardwood	*Fagus sp.*		On a slab and a mesh	cutting tool	in ink	suparallel
Ho / PH / T. 138	Imported hardwood	*Ulmus sp. Minor*		On a slab	/	in ink	196
Ho / PH / T. 141	Imported hardwood ?	*Cf. salix*		On a slab	cutting tool	in ink	96
Ho / PH / T. 162	Local hardwood	*Cf. Ficus ou acacia*	Cambium	On a slab	cutting tool	engraved	82
Ho / PH / T. 170	Imported conifers	Cf. *Pinus*		On a slab	cutting tool	in ink	217
Ho / PH / T. 172	Imported conifers	*Cedrus* sp.		On a slab	cutting tool	in ink	191
Ho / PH / T. 182	Imported conifers	Cf. *Pinus*		On mesh	cutting tool	in ink	suparallel
Ho / PH / T. 184	Imported conifers	Cf. *Pinus*		On a slab and a mesh	cutting tool	in ink	468
Ho / PH / T. 199	Imported hardwood	*Fagus sp.*		On mesh	cutting tool	in ink	suparallel
Ho / PH / T. 210	Imported hardwood	*Fagus sp.*		On a slab	cutting tool	engraved	310
Ho / PH / T. 211	Imported conifers	Cf. *Pinus*		On mesh	/	engraved	374
Ho / PH / T. 225	Imported conifers	Undetermined		On a slab	/	engraved	127
Ho / PH / T. 226	Local hardwood	*Tamarix sp.*		On a slab	cutting tool	engraved	226
Ho / PH / T. 229	Imported hardwood	*Fagus sp.*		On a slab	/	in ink	246
Ho / PH / T. 255	Local hardwood ?	Undetermined		On a slab	/	in ink	86

Table 3 : Description of the various data relating to the mode of cutting, the traces of tools and the estimate of the sizes.

Some arrangements, difficult to discriminate based on visual examination, are perfectly visible on the XRCT images. A hole with its peg still in place is, for example, observed in label HO108 (Fig 6). The shape and undulations of the peg hole are clearly visible on the XRCT images, often suggesting that it was drilled by a spoon drill. Finally, the XRCT images allow us to confirm that some inscriptions were carved into the wood with small chisels or punches. It was also possible to determine the number of chisel strokes required and the depth of the cuts (see, for example, labels HO162 and HO226 in Fig 6). Detailed analysis of the engraved inscriptions on label HO226 shows that all Greek letters, except for "pi" and "mu" (4 mm chisel)

were made with a minimum of strokes using an 8 mm chisel (Fig 7). Some letters required at least two chisel strokes in the extension of the first one to form the longest strokes, such as the bars of "nu" or the sides of "alpha". The letters engraved in the center of the label and on the edges have a different depth (2.5 and 1 mm).

Figure 7 : Analyses of the depths of the multiple chisel strokes in the label to make the inscription and details of the number of the two chisels used.

Timber size estimation

Original wood size were estimated from XRCT images from the convergence of woody rays and/or ring curves. For three labels, HO18, HO61, and HO162, the presence of cambium or bark was used to accurately determine the size of the tree used. For most of the labels, however, the outermost portions of the original tree were absent, and the size provided here has to be seen as a minimum estimate. Finally, for labels HO33, HO58, HO95, HO106, HO137, HO182, and HO199, which were derived from radial cuts, the low curvature of the growth rings did not allow any estimation of tree size.

For the 31 usable labels, the minimum diameters of the trees used for mummy label production ranged from 44 to 468 mm (Table 3). Despite the small sample size, a distinction can be made between imported broadleaved, endemic broadleaved and imported conifer species. The sizes of imported conifers (94-468 mm, avg. 244 mm, n=8) were significantly larger than those of imported broadleaves (70-310 mm, avg. 159 mm, n=16) and especially of endemic broadleaves (44-314 mm, avg. 133 mm, n=7) (Fig 8) These results must be qualified, however, by the method of cutting. Cutting on a slab from endemic broadleaves, imported broadleaves and conifers, labels had average diameters of 133, 159 and 157 mm respectively. The conifer labels made on mesh are from trees with larger diameters (average 284 mm).

Figure 8 : Distribution of mummy labels by cutting method and tree family type, as well as estimated tree sizes used to make the labels.

Comparison of dendrochronological acquisition methods

In general, the methods of acquisition of tree-ring width series vary according to the visibility of growth rings. Measurements based on photographs is optimal when the rings on the face or the flat side of objects were wide and easily identifiable. When the rings were too narrow, the use of a measuring table was more appropriate. In the case of labels, the implementation of these two methods was sometimes complicated by (i) ring boundaries that were difficult to identify, (ii) rings that were too thin (e.g., in the case of label HO106 for which average ring width was 0.24 mm) or (ii) by the presence of a coating (e.g., paint, wax, grease, patina of use) or inscriptions (engraved or inked) (see e.g. HO211) In these cases, XRCT imaging was the only non-invasive acquisition method to accurately delineate the rings and to measure their widths.

To analyze in detail the advantages and disadvantages of the different methods, we compared the series of ring widths obtained from photographs or thanks to the measuring table made on the flat side of the labels with those measured on XRCT images on the transverse plane. As a general rule, and with the exception of label HO182 (a very small difference when scaled to 1/100th of a mm), the traditional measurements were superior to those made on XRCT images. Differences between methods, however, only rarely exceeded a few 0.01 mm and depended mainly on the cutting. In the case of radial (on mesh) cutting (see e.g. labels HO43, HO85, HO182 and HO184), which was close to the radial growth of the rings, differences were very small (Fig 9). The discrepancy between the acquisition methods was maximal for cutting on slabs or close to the pith (e.g., HO172 and HO170). In the case of HO170, the traditional methods exceeded on average by 117% the widths measured on XRCT images. However, this difference was heterogeneous and was more important in the pith or in the first growth rings, and also varied according to the orientation and curvature of the rings.

Figure 9 : Comparison of the differences in ring width values between those made on the label faces with those made from the CTs.

Discussion

In this paper, 38 mummy labels were selected from the BNU Strasbourg collection and analyzed with X-ray computed tomography (XRCT) imagery. We evaluated the potential of XRCT images for anatomical identification, identification of tool marks, determination of wood size and measurement of ring widths and compared results with traditional visual observations or optical (binocular, microscope) and digital (high resolution photography) analyses.

Contribution XRCT imaging for anatomical identification

XRCT imaging allows more precise insights into the morphology of certain anatomical markers such as wood rays or distribution, shape and number of pores, but also to better visualize ring boundaries. However, these analyses are limited to the transverse plane because the resolution of XRCT images is too low to properly assess anatomical markers in the tangential and radial planes. Magnifications of x100 to x400 would be required to facilitate recognition of anatomical markers [32], which is well beyond current resolutions of XRCT. This limitation prevents formal identification of most species except for a few imported broadleaves with ring-porous zones (elm) or diffuse pores (beech, willow). For local species, XRCT observations suggest that some labels are made of tamarisk, fig, acacia, or jujube, but at definitive identification would only be possible by measuring the widths and heights of woody rays in the tangential and radial planes [34,35]. For conifers, the presence of resin ducts and the transition from earlywood to latewood was used on XRCT images to differentiate species groups but not for specific identification. Despite the benefits of tomography, species detection is not possible without the removal of a small piece of the label [9].

A corpus of 420 mummy labels with identified species, from the collections of the British Museum (n=250, https://www.britishmuseum.org/collection/), the Louvre (n=122, https://collections.louvre.fr/; [43], and the Mediterranean Archaeology Museums of Marseilles

(n=44) and Amiens (n=4) [29], supports some of our hypotheses (Fig 10). In particular, it confirms the existence of labels made of cedar (*Cedrus libani* or *atlantica*, n=77, 41% of conifer labels) and pine (n=68, 36%). Whereas cypress (n=8, 4%) and juniper (n=10, 5%) are much less represented, the proportions of species conserved in these museums seem to be consistent with our results (n=1 for either of the species).

Figure 10 : Taxonomic variety of different species identified for mummy labels from the collections of the British Museum, the Louvre, the Musée de Picardie in Amiens and the Musée Archéologique de Marseille.

For imported and local broadleaved species, any validation of our hypotheses is less obvious. Beech (*Fagus* sp.) is well represented in the collections (n=13, or 46% of the imported broadleaves) as it is at the BNU in Strasbourg. The single elm (*Ulmus* sp.) label is attested by only one other occurrence in the British Museum collection (n=1, 3.5%). In addition, the presence of a single row of pores in the well-characterized pore zone of label HO128 and wood rays that are 2-5 cells wide would clarify the identification to the mounting elm (*Ulmus glabra*) type [36].

In the case of the endemic species, fig (*Ficus sycomores*, n=44, 27.5% of local broadleaves), tamarisk (*Tamarix* sp., n=66, 41%) and jujube (*Ziziphus spina-christi*, n=37, 23%) are well represented in our analyses (n=3 for fig, n=7 for tamarisk, n=1 for jujube) and the corpus of 420 labels. On the other hand, we identified two acacia labels, whereas this species only represents 4% of the labels from local broadleaves, which may possibly call our initial attribution into question as it was indeed sometimes quite complex to distinguish acacia and fig samples based on the transverse plana only due to rather similar anatomical distinction keys.

Finally, willows (*Salix* sp.) are fairly well represented in the corpus (n=27, 59% of imported and/or local broadleaved species) and in our analyses (n=2). However, the complex

identification of this diffuse-porous species in no way allows for a definitive attribution. Furthermore, the broad taxonomic spectrum [32,35,36] includes more than ten different willow species for the two willow labels in the corpus. Some species, such as the Egyptian willow (*Salix subserrata*) identified on a mummy label from the Louvre [43] is endemic to Egypt, yet a the distinction between imported and endemic deciduous is therefore impossible on the basis of XRCT images.

Technological and dendrological interpretations from XRCT images

For the majority of the clear and visible tool marks, the added value of XRCT as compared to observations under a binocular is relatively limited. In both cases, the distinction is limited to the traces of saws and cutting tools such as axes, adzes, planes, chisels and planes, all of which have been employed in ancient or Roman times [28,37,44,45]. The interest of XRCT is, however, undeniable for the detection of faint or invisible tool marks. In the case of the peg hole of label HO108 (Fig 6), for instance, tomography allows to confirm a reuse of the wood. That is, the pointed shape and undulations of the peg hole, clearly visible on the XRCT images, also suggest that it was drilled from a spoon bit, a tool being known both in the Roman Empire [46,47] and in ancient Egypt [48-49]. Similarly, detailed analysis of the engraved inscriptions on label HO226 opens up new insights into the gestures and practices of woodworking skills. The varying depths of the Greek letters, for example, may be related to a slight concavity of the label which ensures a good adhesion of the central part on the working surface but renders the chiseling more complex on the edges. The minimal number of chisel strokes also suggests, even if this hypothesis will have to be validated by replication on a larger dataset, that this inscription was made by a person (e.g., craftsman, scribe) with a perfect mastery of woodworking.

The added value of XRCT images in highlighting cutting methods and estimating sizes is especially important at the level of the ends of labels, which are often only poorly legible. Still underdeveloped [12,14], this approach is crucial as it can help to better information on collection practices in a wood-importing country due to wood resources that are limited to the Mediterranean coast, the Nile Delta, the banks of the Nile and the main oases [50]. Despite the limitations of the limited sample size, our analysis shows correlations between the cutting methods used, the size of trees and the species used to produce the mummy labels. These results show that the largest diameters estimated (on average 245.5 mm) are most often cut on mesh (probably by splitting) from conifers (n=5). In the case of some labels (n=7) with radial cutting, an estimation of the diameter was not possible because of the subparallel aspect of the wood rays and the weak curvature of rings, but in any case these labels were cut from large caliber trees [39,51]. Medium- to small-sized calibers (135 mm on average) are most often cut on a slab by sawing local broadleaved species. In the absence of traces corresponding to a length sawing, it is reasonable to think that the latter was carried out by means of a handsaw, known as a "pull saw", widely used in ancient Egypt [48] but not very adapted for large ~~calibers.~~ It should be noted, however, that reworking of wood, very common in Egypt [15], likely biases interpretations, as an initial cutting on a mesh can be reworked later with a saw, especially in the case of dry wood. Finally, the frequent use of saws on local woods indicates that these woods, which are gnarled and very dense due to rapid and continuous growth, are not suitable for cutting by splitting due to the lack of a preferential splitting plane. Different craft practices are therefore used depending on the origin of the wood exploited and the tools used.

Comparison of XRCT and traditional dendrochronological measurements and the dendrochronological potential of species endemic to Egypt

The measurement of ring widths made using the three methods of acquisition (measurement based on images, classical ring-width measurements on a table on the flat side of the mummy labels or using XRCT images) show significant differences, depending on the cutting mode. These differences are maximal in the case of cutting on a slab close to the pith (HO170 and 172). Despite the small size of our sample, these differences show that some dendrochronological analyses made on the flat side of other mummy label collections are potentially biased. However, the absence of a reference for the eastern Mediterranean basin in Antiquity does not allow quantification of the impact of these biases on sample dating. Regarding inter-series synchronization, only labels HO182 and HO184 show Student's T values >5 and r correlation values >0.7 for an overlap of 28 years, suggesting contemporary woods. For these two timbers, correlations obtained between the series of widths measured on XRCT images (r=0.764, T=5.3) are higher than those obtained between XRCT and traditional tree-ring measurements (r=0.717 and 0.763, T=5 .151 and 4.824) or between traditional dendrochronological measurements (r=0.715, T=4.691). These results obviously need to be replicated, but suggest an added value of XRCT for the measurement of tree-ring widths, synchronization and dating of the series, most certainly due to perfectly oriented measurements along the transverse plane.

Tomographic imaging also confirmed the limited dendrochronological potential of endemic species. For the construction of dendrochronological reference chronologies, distinct, annual rings and a sufficiently large numbers of rings allowing synchronization are needed [52]. None of the species identified with XRCT appear to meet these criteria for the time being. XRCT analyses did neither allow identification of ring boundaries in sycamore (*Ficus sycomorus*) or common (*Ficus carira*) fig labels, nor in the acacia labels. Consistent with observations by Kuniholm *et al.* [17], Creasman [13] and El Sherbiny [53], the parenchyma bands visible in the XRTC images are not, in the case of these species, ring boundaries. For fig, this is confirmed by the distribution of pores, which are either isolated or radially abutting, mostly distributed between fiber and parenchyma bands. The seven labels potentially made of

tamarisk and the label produced from jujube sometimes show distinct rings, as described by Gale & Cutler [34], but the legibility of rings is very heterogeneous and the number of rings is often below 20. This is in line with observations made on living trees in Africa and the Near East on several species, including acacia and tamarisk [54-62], showing rapid growth and short series that are difficult to use for the elaboration of dendrochronological references. Therefore, imported conifers and broadleaved species remain the best candidates for the construction of reference chronologies for this part of the Mediterranean [17,19,63-65].

Conclusions

The results obtained fom 38 mummy labels from the BNU in Strasbourg confirm the potential of X-ray computed tomography (XRCT) for the analysis of archaeological material [9]. Even if species could not be formally identified due to the insufficient resolution of the XRCT images on the longitudinal planes [9], the results obtained on the transverse planes show that 11 labels are probably from imported conifer, 10 from imported broadleaved and 17 from local broadleaved species.

Tomography is not indispensable for the recognition of tool marks. It does, however, undeniably improve observations of the frequency and depth of tool blows, highlighting developments invisible to the naked eye and providing an unambiguous view on cutting methods. It constitutes, therefore, an innovative approach to provide new information on the silvicultural practices and skills of Egyptian craftsmen.

As far as the dendrochronological approach is concerned, tomography, by its non-invasive character, appears particularly adapted to the analysis of museum collections. XRCT images of the transverse plane offer a clear view of possible ring boundaries [7-9] that are often difficult to observe and measure using traditional methods. XRCT therefore allows insights into objects and exploration of the full potential of archaeological material for dendrochronological purposes. Highly-resolved XRCT imagery can thereby also contribute to

the elaboration of solid reference chronologies ~~which~~ can then be used for dating purposes or the analysis of past climate.

In terms of accuracy, measurements made on the flat side of conifer labels from traditional approaches and on the transverse plane of XRCT images do not show significant differences, in the case of radial cutting. In the case of cutting on a slab, differences are sometimes significant near the pith or for the first rings depending on the orientation of the rings, with likely impacts on the synchronization of ring widths. It would be relevant to develop approaches further to correct these differences for measurements made on the flat side of objects.

In terms of potential, tomography confirms the limited dendrochronological potential of several local species (fig, tamarisk, acacia, jujube) as they do not show distinct rings, rarely visible rings, or only a small number of rings (<20) insufficient for cross-dating and the construction of robust dendrochronological reference chronologies. Although other approaches could be tested (such as quantitative wood anatomy, or QWA) to increase the dendrochronological potential of these local species, our results suggest that future work should focus on imported conifer labels, which are easier to measure radially and likely to provide longer series [15].

Acknowledgments

The tomographic analyses carried out on 38 mummy labels was financed in the framework of the SNSF project n° 192176 "The Roman Egypt Laboratory: Climate Change, Societal Transformations, and the Transition to Late Antiquity". We warmly thank the engineers of the Institut Charles Sadron, CNRS - UPR22 of Strasbourg, Damien Favier and Antoine Egele, for their professionalism and for taking the time to process all the selected mummy labels. We would like to thank Véronique Asensi Amoros for her advice concerning anatomical identifications and the exploitation of tomographic images.

References

1. Lavier C. Archéodendrométrie sur objets et œuvres d'art à support-bois : savoir-faire technique, capacités, obstacles et alternatives. Sciences analytiques, 2013; *292:* 67-73.

2. Guibal F. Dendrochronologie des épaves de navires antiques de Méditerranée. Archaeonautica, 1998; 14: 303-308.

3. Gassmann P, Lambert G, Lavier C. Pirogues et analyses dendrochronologiques. In : Arnold B., Pirogues monoxyles d'Europe centrale: construction, typologie, évolution, tome 2. Neuchâtel: Musée cantonal d'archéologie; 1996. pp. 89-127.

4. Thomas E, Blondel F, Colson I, Feyburger C, Tran QK, Les ex-voto en bois gallo-romains de la Source des Roches de Chamalières (Puy-de-Dome). Vers une compréhension de leur évolution structurale pour une meilleure conservation. Revue Conservation Restauration des biens culturels, 2016; 34: 27-39.

5. Blondel F, Bèche-Wittmann M, Dacko M, Dousteyssier B, Gaumat C, Jamois T *et al.* Terminus ! Énigme partiellement résolue. In: Bet P, Dousteyssier B directors, Eclats Arvernes II, Fragments archéologiques (Ier - Ve siècle apr. J.-C.). Clermont-Fd: Presses univ. Blaise Pascal, MSH; 2021. pp. 462-465.

6. Fraiture P. Contribution of dendrochronology to understanding of wood procurement sources for panel painting in the former Southern Netherlands from 1450 AD to 1650 AD. Dendrochronologia, 2009; 27 (2): 95-111.

7. Dominguez-Delmas M, Bossema GF, Dorscheid J, Coban SB, Hall-Aquitania M, Batenburg J, *et al.* X-Ray computed tomography for non-invasive dendrochronology reveals a concealed double paneling on a painting from Rubens'studio. PLoS ONE, 2021; 16(8).

8. Bossema FG, Dominguez-Delmas M, Palenstijn WJ, Kostenko A, Dorscheid J Coban SB *et al*. A novel method for dendrochronology of large historical wooden objects using line trajectory X-ray tomography. Nature, portfolio, scientific report, 2021.

9. Grabner M, Salaberger D, Okocki T. The need of High Resolution u-X-ray CT in Dendrochronology and in wood Identification. In: ISPA 2009 - Proceedings of the 6th International Symposium on Image and Signal Processing and Analysis*:* 2009*.* pp. 359-362.

10. Conchon P, Badel E. La microtomographie 3D. Quand l'imagerie X permet d'explorer la structure des végétaux. 15e journée de la mesure et de la métrologie, oct. 2018, St-Pierre d'Oléraon, France. J2M2018; 2018. pp. 102.

11. Fraiture P, Weitz A. Aperçu des résultats de l'étude dendroarchéologique des cercueils en bois du XIIe siècle provenant de l'Abayye de Nivelles (Belgique, Brabant wallon). In: Shindo L, Edouard JL, Suméra F, Bailly M, Hartmann-Virnich A directors. ARCADE. Approche diachronique et Regards croisés : Archéologie, Dendrochronologie et Environnement. Aix-en-Provence : Drac de Provence-Alpes-Côte d'Azur, 2019. pp. 117-130.

12. Girardclos O, Petit C. Dendro-archaeological contribution to the history of forest exploitation: a case of the Gallo-Roman settlement of Oedenburg (Alsace, France) between 10 and 180 AD. In: Fraiture P. Proceedings of an international conference, Tree Rings Art Archaeology, Royal Institute for Cultural Heritage; 2011. pp.77-97.

13. Creasman PP. The potential of dendrochronology in Egypt: Understanding Ancient Human / Environment Interactions. In: Ikram S, Kaiser J, Walker R editors. Egyptian Bioarchaeology, humans, animals and the environment. Leiden: Sidestone Press; 2015. pp. 201-210.

14. Blondel F, Labbas V, Shindo L. La dendrochronologie : potentialités et nouveaux enjeux pour l'archéologie préventive. In: Carpentier C, Arbogast RM, Kuchler P directors. Bioarchéologie: minimum méthodologiques, référentiels communs et nouvelles approches,

actes du 4e séminaire scientifique et technique de l'Inrap, 28-29 novembre 2019, Sélestat. 2020.

15. Blondel F, Huebner SR, Pearson C, Markus S. Mummy labels: a Witness to the Use and Processing of Wood in Roman Egypt. IJWC, 2023.

16. Kuniholm PI, B Kromer SW, Manning M, Newton CE, Bruce L, Bruce MJ. Anatolian tree rings and the absolute chronology of the eastern Mediterranean, 2220–718 BC. Nature. 1996; 381: 780–783.

17. Kuniholm PI, Newton M, Sherbiny H, and Bassir H. Dendrochronological Dating in Egypt: Work Accomplished and Future Prospects. Radiocarbon, 2014; 56 (04): 93–102.

18. Creasman PP. Tree Rings and the Chronology of Ancient Egypt. Radiocarbon, 2014; 56 (04): 85–92.

19. Creasman PP, Dean J S, Babst F. Eine Einführung in die Dendrochronologie für Ägyptologen. Göttinger Miszellen, 2015; 245: 49-66.

20. Bornemann D. Les collections égyptologiques et papyrologiques de la Bnu. La Revue de la BNU [Online], 2, 2010.

21. Spiegelberg W. Aegyptische und griechische Eigennamen aus Mumienetiketten der römischen Kaiserzeit, auf Grund von grossenteils unveröffentlichtem Material gesammelt und erläutert. Leipzig: J.C. Hinrichs; 1901.

22. Vleeming SP. Demotic and Greek-Demotic mummy labels and other short texts gathered from many publications (short texts II 278-1200), Leuven - Paris: Peeters; 2011.

23. Gaudard F, Martin Hernandez R, Torallas Tovar S. Mummy label Database (MLD). The oriental institute, Annual Report; 2008-2009: 96-100.

24. Quaegebeur J. Mummy labels: An Orientation. In: E Boswinkel, PJ Pestman editors. Textes grecs, démotiques et bilingues. Papyrologia Lugduno-Batava,1978; 19: 232–259.

25. Worp KA. Greek, Greek/Demotic and demotic mummy labels: A survey, Death on the Nile Online Publications, Version 2.0. March 2017.

26. Firon F. La mort en Égypte romaine. De l'encadrement par le pouvoir romain à la gestion personnelle (de 30 av. J.-C. au début du IVe siècle apr. J.-C.). SilvanaEditoriale, Milan, 2020.

27. Lucas A, Harris JR. Ancient Egyptian, materials & industries. 3e ed. London: Edward Arnold publishers LTD, 1948.

28. Gale R, Gasson P, Hepper N, Killen G. Wood. In Nicholson, PT, Shaw I editors. Ancient Egyptian Materials and Technology, Cambridge: Cambridge University Press, 2000.

29. Asensi Amoros MV. L'étude du bois et de son commerce en Égypte : lacunes des connaissances actuelles et perspectives pour l'analyse xylologique. In: Neumann K, Butler A, Kahlheber S directors. Food, Fuel and Fields: Progress in African Archaeobotany. Köln: Heinrich – Barth institut, 2003. pp. 177-186.

30. Cartwright CR. Understanding Wood Whoices for Ancient Panel Painting and Mummy Portraits in the APPEAR Project through Scanning Electron Microscopy. In: Svoboda M., Cartwright C. editors. Mummy Portraits of Roman Egypt. Emerging Research from the APPEAR Project. Los Angeles: J. Paul Getty Museum; 2020. pp. 14-23.

31. John J. Variation of wood anatomy in relation to environmental factors in two southern African broadleaves. Departement of Pure and Applied Biology, imperial College, London, Unpublished PhD Thesis; 1990.

32. Schweingruber FH. Anatomie microscopique du bois. Editions Fluch-Wirth Zurich; 1990.

33. Schweingruber FH. Anatomy of European woods. Verlag Paul Haupt, Bern and Stuttgart; 1996.

34. Gale R, Cutler D. Plants in archaeology. Identification manual of artefacts of plant origin from Europe and the Mediterranean. Westbury and Royal Botanic Gardens, Kew, West Yorkshire; 2000.

35. Neumann K, Schoch W, Détienne P, Schweingruber FH, Richter HG. Woods of the Sahara and the Sahel: An Anatomical Atlas. Haupt, Bern, Stuttgart and Vienna; 2000.

36. Akkemik Ü, Yaman B. Wood anatomy of Eastern Mediterranean Species. Kessel Publishing House: Remagen-Oberwinter; 2012.

37. Applequist MB. A simple pith locator for us with off-center increment cores. F. For, 1958; 56: 151.

38. Rozas V. Tree age estimates in Fagus sylvatica and Quercus robur: testing previous and improved methods. Plant Ecology, 2003; 167: 192–212.

39. Blondel F, Girardclos O. Approche dendroarchéologique de l'approvisionnement de la ville antique d'*Augustonemetum* (Clermont-Ferrand – Puy-de-Dôme) en bois d'œuvre et exploitation forestière. ArcheoSciences, Revue d'Archéométrie. 2018; 42-1: 17-33.

40. Rinn F. Eine neue Methode zur Messung von Jahrringparametern. Diplomzulassungsarbeit am Institut für Umweltphysik, Universität Heidelberg, September 1986 - February 1988; 1986, 85 pp.

41. Larsson LA. Cybis CooRecorder—Image Coordinate Recording Program & CDendro—Cybis Dendro Dating Program. Cybis Elektronik & Data AB; 2017.

42. Delwaide A, Filion L. Echantillonnage et datation dendrochronologiques (Chapitre 7). In: Payette S, Filion L, La dendrochronologie. Principes, méthodes et applications. Québec: Presses de l'université de Laval; 2010. pp. 167-197.

43. Asensi Amoros MV, Détiennes P. Les bois utilisés pour les portraits peints en Égypte à l'époque romaine. In: Aubert MF, Cortopassi R, Nachtergael G, Asensi Amoros MV, Détienne P, Pagès-Camagna S *et al.* Portraits funéraires de l'Égypte romaine, cartonnage, linceuls et bois. Khéops & Musée du Louvre éditions, Besançon; 2008. pp. 28–40.

44. Bruwier MC. Le travail du bois. In: Bruwier MC, Cession-Louppe J, De Jongle D, Evrard-Derriks C, Gratien B, Kruchten JM *et al.* Artisans de l'Égypte ancienne. Morlanwelz: Musée Royal de Mariemont, 1981. pp. 38-40.

45. Long DA. More Early Planes. An account of thirteen woodworking planes dating from the late second century AD to the eleventh century AD. Bulletin Instrumentum, 2006; 24: 15-18.

46. Tisserand N. Les outils en fer du site de Vertault-Vertillum (Côte-D'Or). In: Chardron-Picault P director. Aspects de l'artisanat en milieu urbain: Gaule et Occident romain. Actes du colloque international d'Autun, 20-22 septembre 2007. Dijon: ARTEHIS éditions (28e suppl. à la RAE); 2010. pp. 251-265.

47. Ulrich RB. Roman Woodworking. New Have, & London: Yale university press; 2007.

48. Bruwier MC, Cession-Louppe J, De Jongle D, Evrard-Derriks C, Gratien B, Kruchten JM *et al.* Artisans de l'Égypte ancienne. Morlanwelz: Musée Royal de Mariemont, 1981.

49. Aldred C. Fine woodwork. In: History of Technology. Oxford; 1954.

50. Deglin F. Wood exploitation in ancient Egypt: where, who and how? In: El Gawad HA, Andrews N, Correas-Amador M, Tamorri V, Taylor J editors. Current research in Egyptology,

Proceedinf of the Twelfth Annual Symposium. Oxbow books, Oxford and Oakville; 2012. pp. 85-96.

51. Marguerie D, Hunot JY. Charcoal analysis and dendrology; data from archaeological sites in north-western France. Journal of Archaeological Science, 2006.

52. Kaennel M, Schweingruber FH. Multilingual Glossary of Dendrochronology. Terms and definitions. Paul Haupt, Birmensdorf; 1995.

53. El Sherbiny HAA. Studies in dendro-Egyptology: the laboratory of tree-ring research Egyptian wooden collection. Thesis of Faculty of the Departement of Geosciences, Master of Science, University of Arizona; 2015.

54. Gourlay ID. Growth ring characteristics of some African Acacia species. Journal of Tropical Ecology, 1995; 11: 121-140.

55. Gourlay ID. The definition of seasonal growth zones in some African acacia species – A review. IAWA Journal, 1995; 16(4): 353-359.

56. Eshete G., Staehl G. Tree rings as indicators of growth periodicity of acacias in the Rift Valley of Ethiopia. Forest Ecology and Management, 1999; 116: 107-117.

57. Touchan R, Hughes MK. Dendroclimatology in the Near East and the Eastern Mediterranean Region. In: Manning SW, Bruce MJ editors. Tree-Rings, Kings and old world Archaeology and Environment. Papers presented in honour of Peter Ian Kuniholm. Oxbow Books, Oxford: 2009, 65–70.

58. Nicolini G, Tarchiani V, Saurer M, Cherubini P. Wood-growth eones in Acacia seyal Delile in the Keita Valley, Niger: Is there any climatic signal? Journal or Arid Environments, 2010; 74(3): 355-359.

59. Wils THG, Robertson I, Eshetu Z, Koprowski M, Sass-Klaassen U G W, Touchan R et al. Towards a reconstruction of Blue Nile Baseflow from Ethiopian tree-rings. The Holocene, 2010; 20(6): 837-349.

60. Wils THG, Sass-Klaassen UGW, Eshetu Z, Bräuning A, Gebrekirstos A, Couralet C, *et al.* Dendrochronology in the dry tropics: the Ethiopian case. Trees, 2011; 25(3): 345-354.

61. Gebrekirstos A, Mitlöhner R, Teketay D, Worbes M. Climate-growth relationships of the dominant tree species from semi-arid savanna woodland in Ethiopia. Trees, 2008; 22: 631-641.

62. Gebrekirstos A, Bräuning A, Sass-Klassen U, Mbow C. Opportunities and applications of dendrochronology in Africa. Current Opinion in Environmental Sustainability, 2014; 6: 48-53.

63. Cichocki O, Bichler M, Firneis G, Kutschera W, Wolfgang M, Stadler P. The synchronization of Civilizations in the Eastern Mediterranean in the Second Millenium BC: Natural Science Dating Attempts. In: Buck CE, Millard AR editors. Tools for Constructing Chronologies, Crossing Disciplinary Boundaries. London: Springer Science & Business Media; 2004. pp. 83-109.

64. Cichocki O. Libanesische Zedern als Datierungswerkzeug in der ägyptischen Archäologie. In: Czerny E., Hein I, Hunger H, Melman D, Schwab A editors. Timelines, Studies in honour of Manfred Bietak, OLA 149, Bd. 1-3; 2006. pp. 293-299.

65. Touchan R, Meko DM, Anchukaitis KJ. Dendroclimatology in the Eastern Mediterranean. Radiocarbon, 2014; 56 (04): 61–68.

Figure 1

Click here to access/download;Figure;Figure1FB.tif

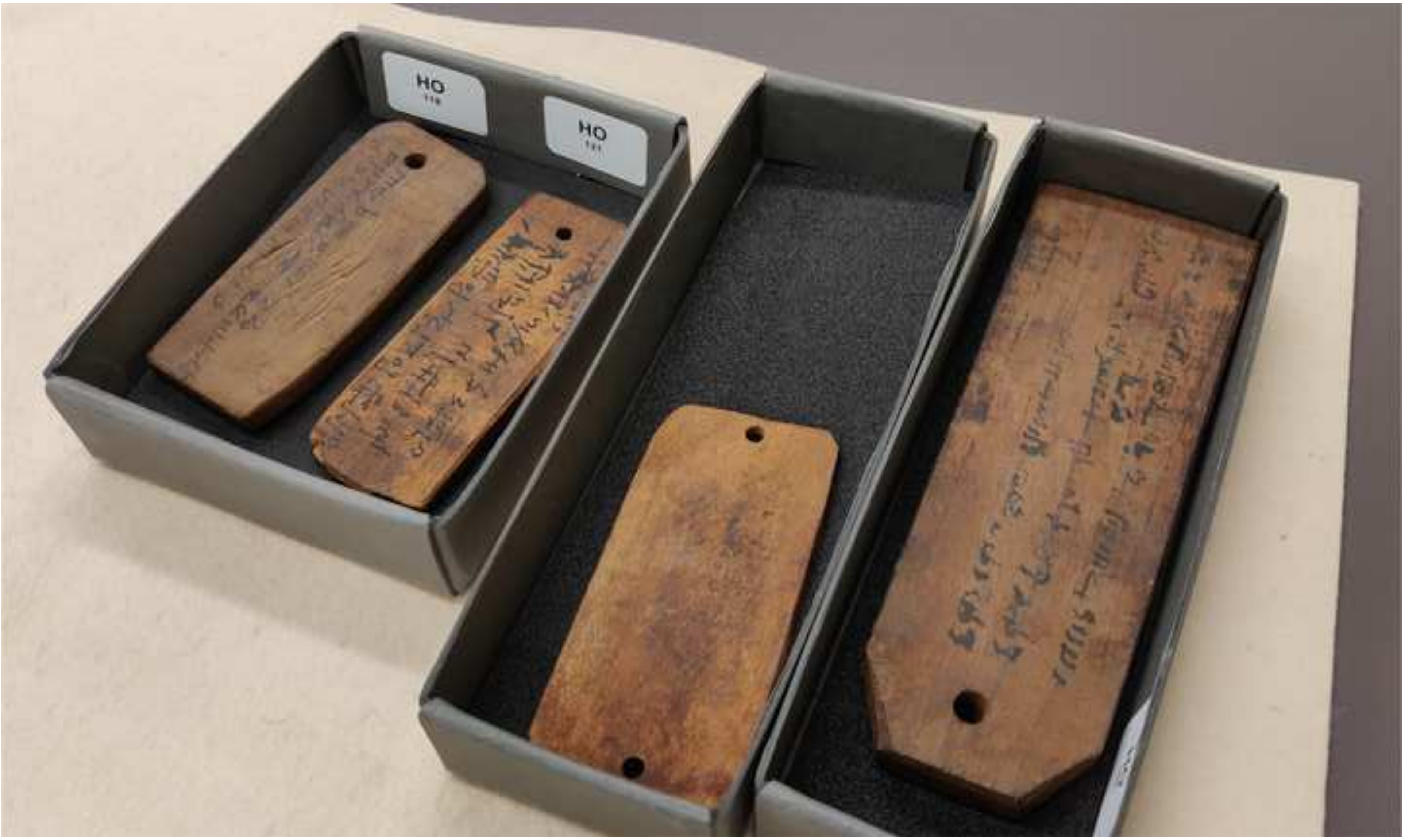

Figure 3

Click here to access/download;Figure;Figure3FB.eps

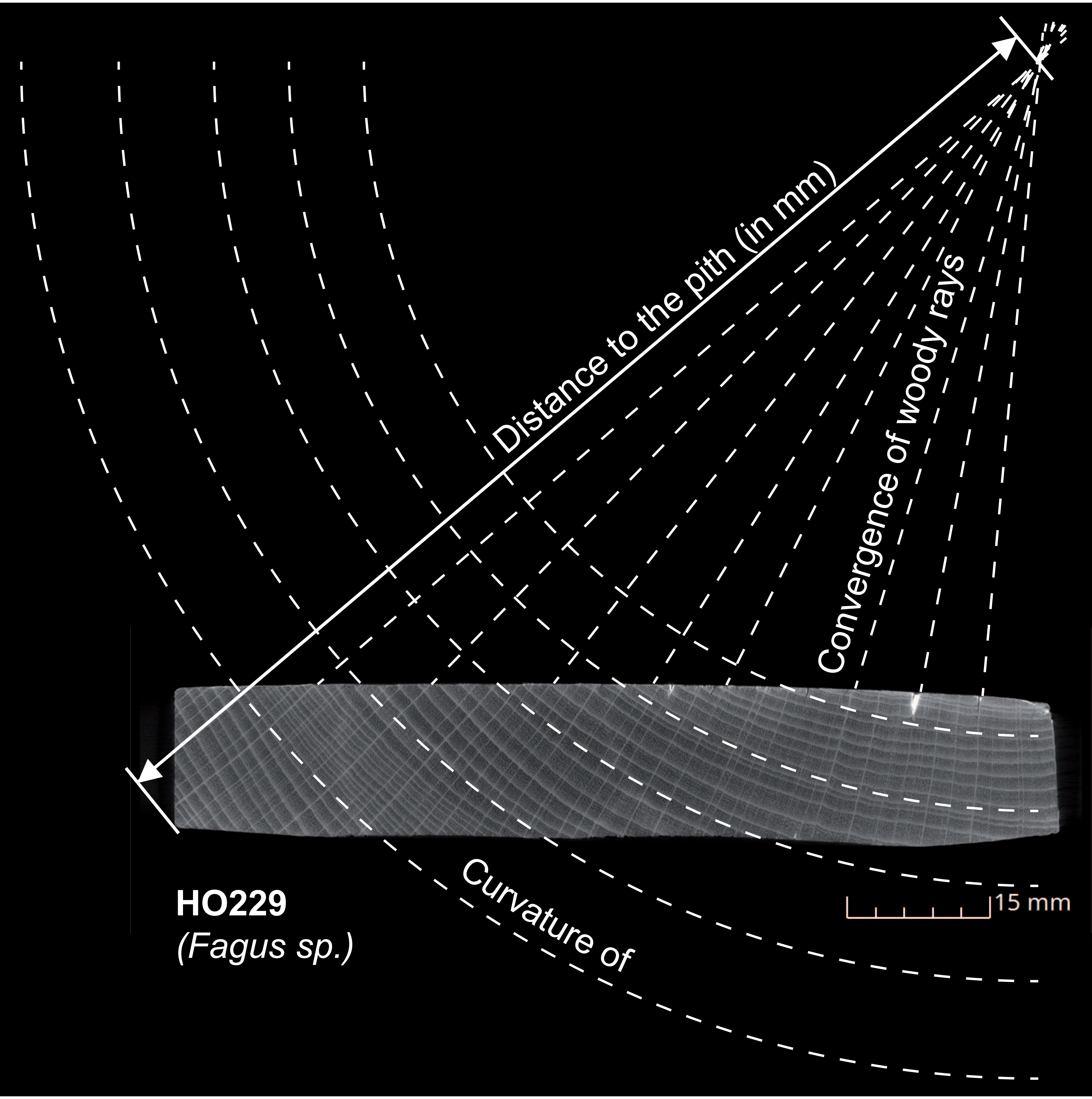

Figur[illegible] Click here to access/download;Figure;Figure5FB.eps

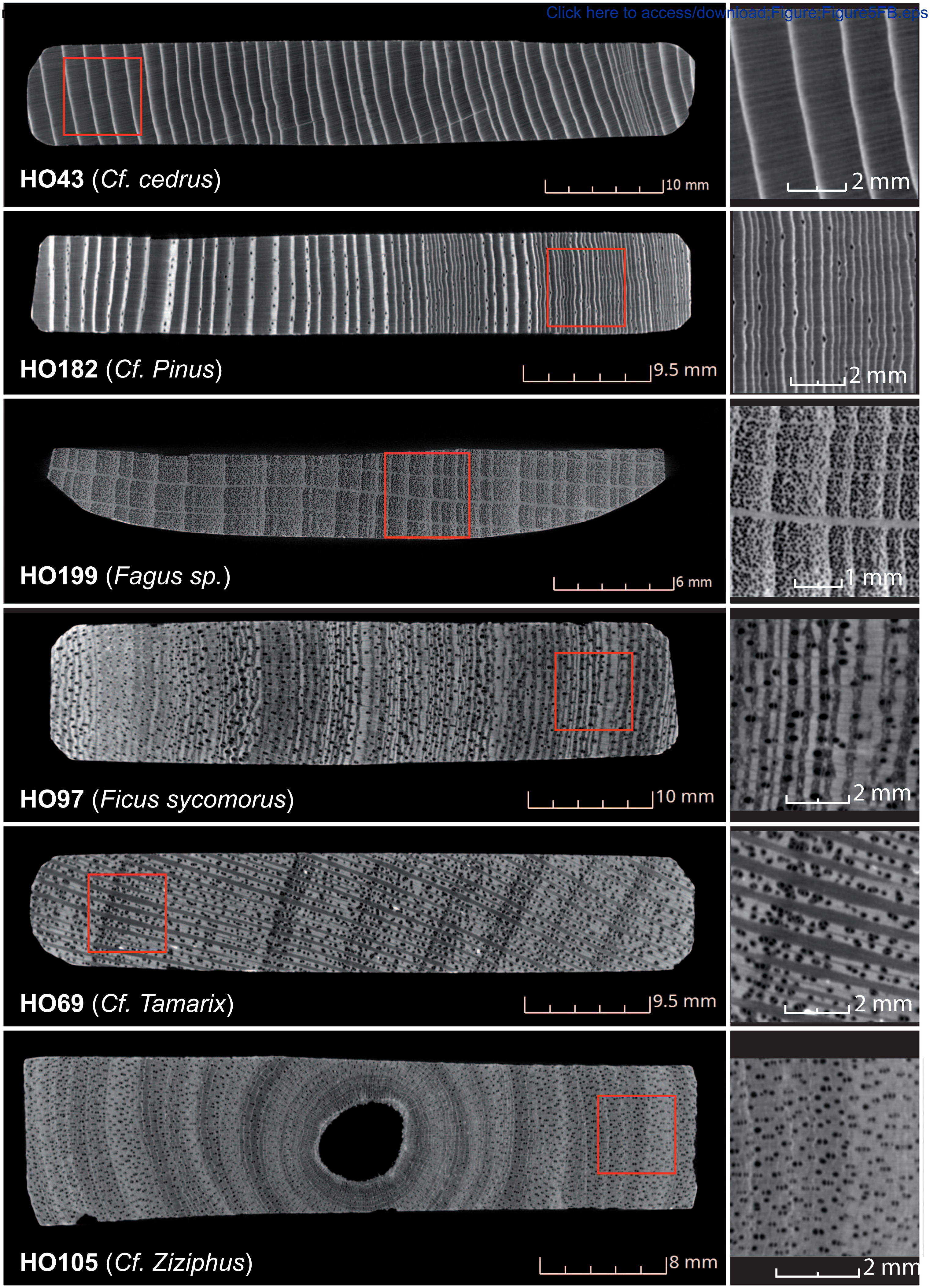

Figure 6

Click here to access/download;Figure;Figure6FB.eps

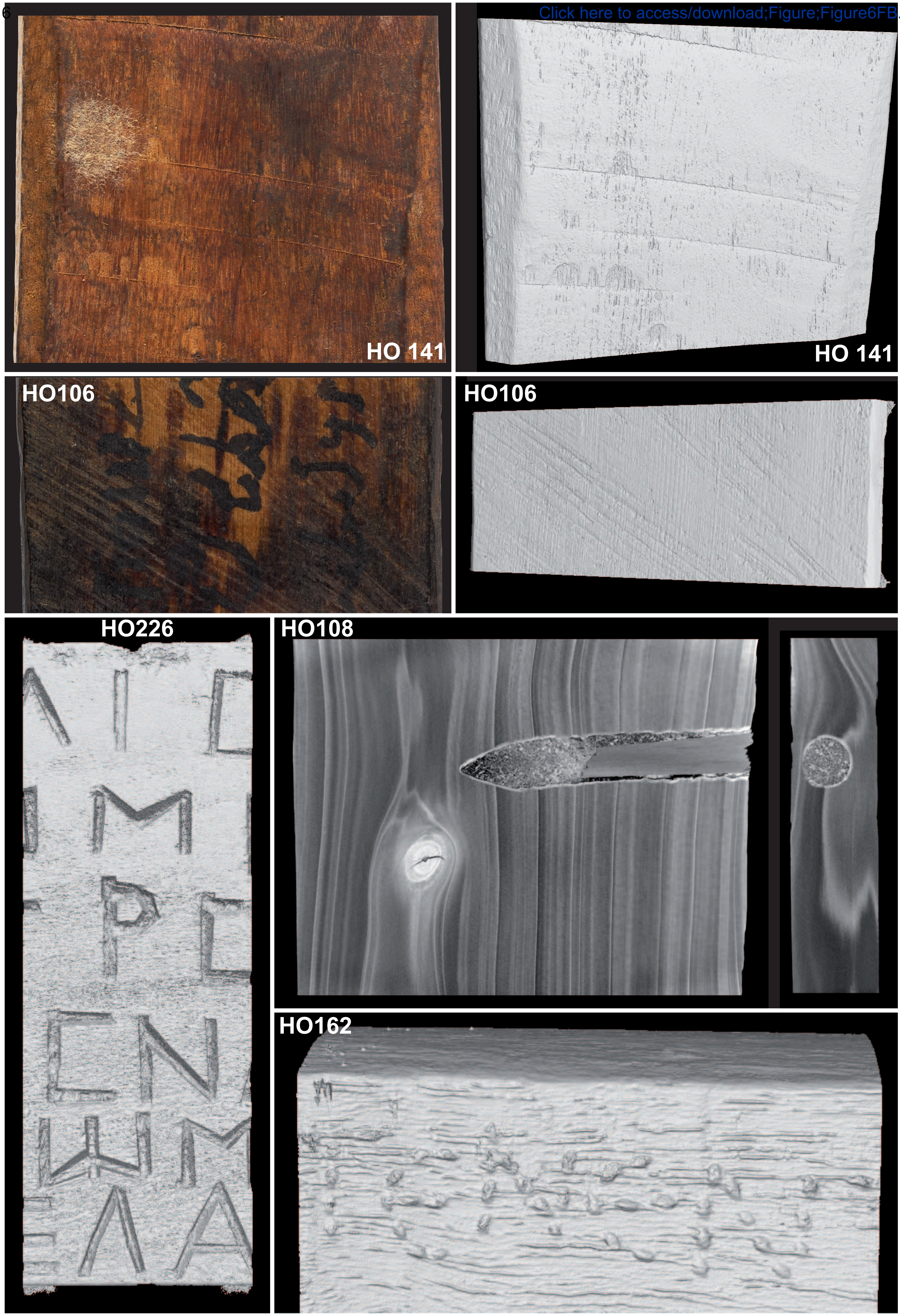

Figure 7

Click here to access/download;Figure;Figure7FB.eps

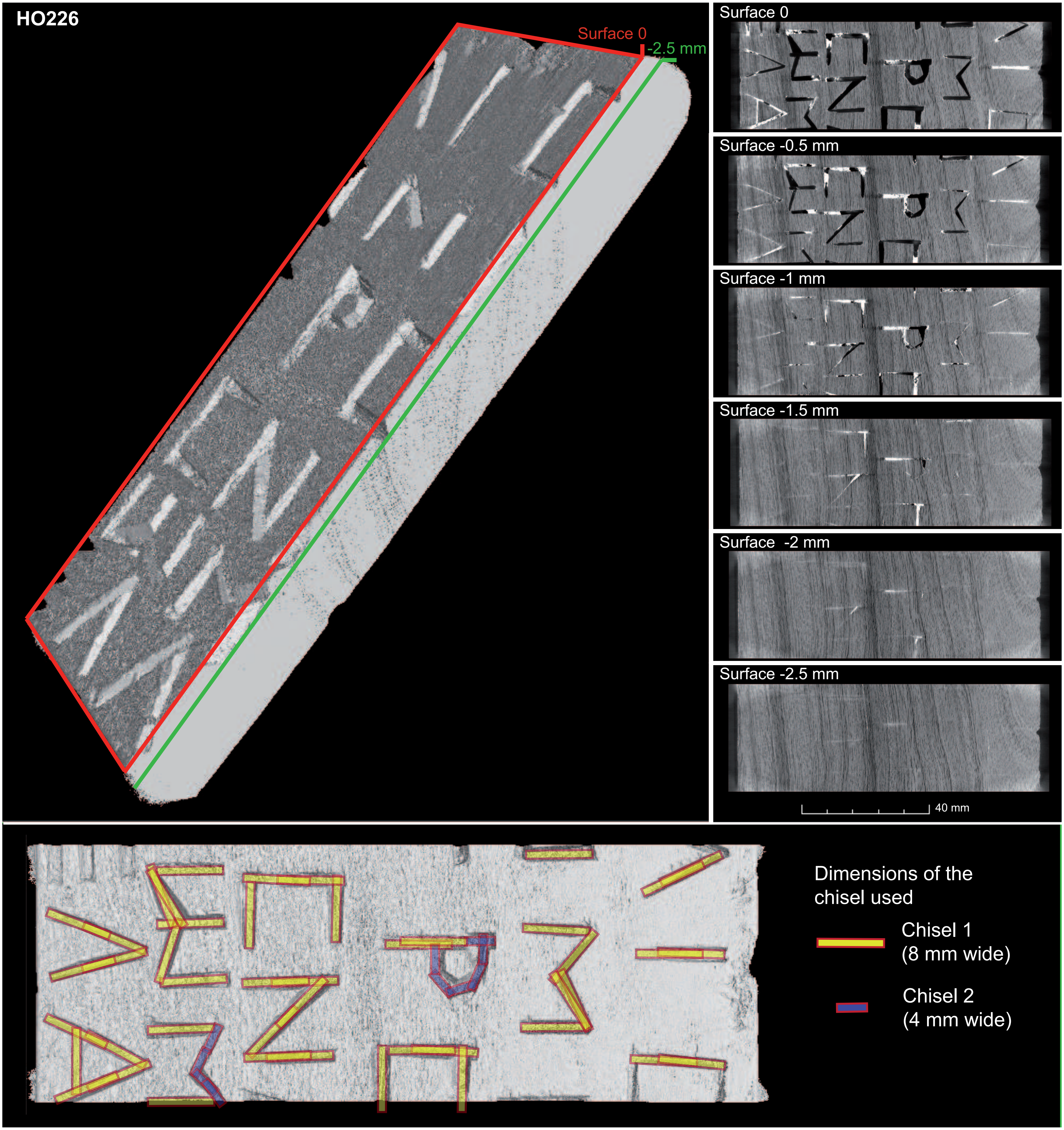

Figure 8

Click here to access/download;Figure;Figure8FB.eps

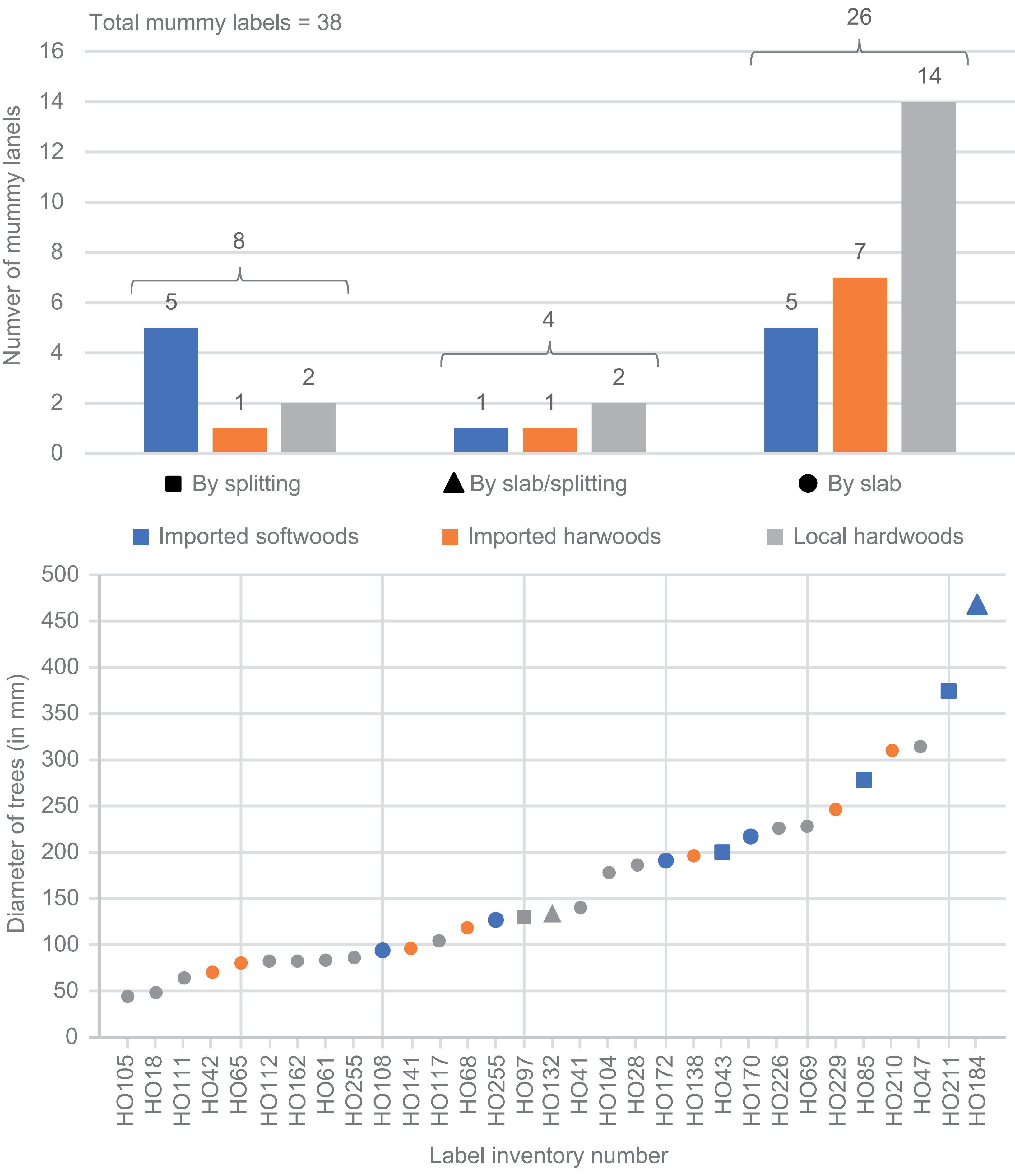

Figure 9

Click here to access/download;Figure;Figure9FB.eps

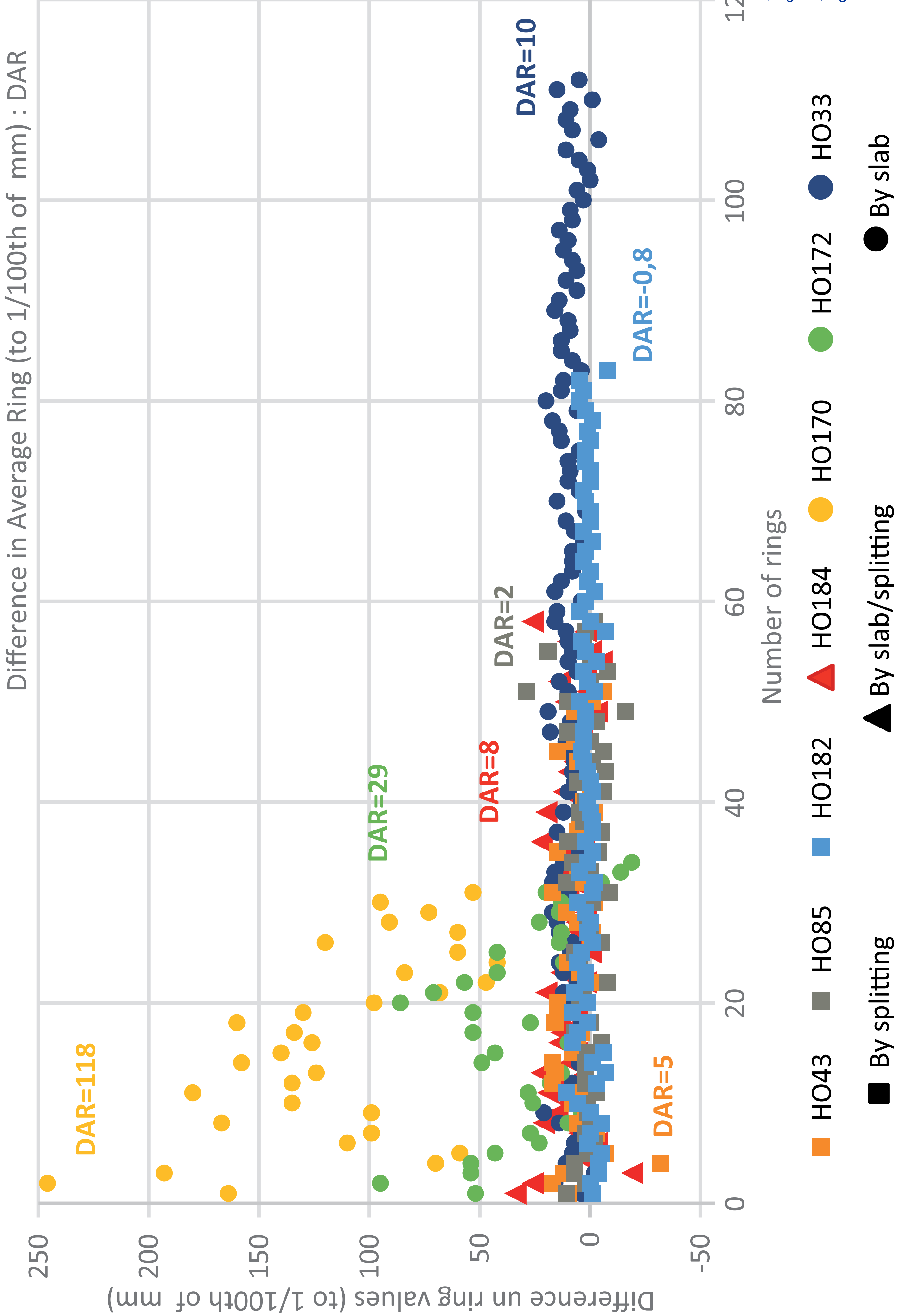

Figure 10

Click here to access/download;Figure;Figure10FB.eps

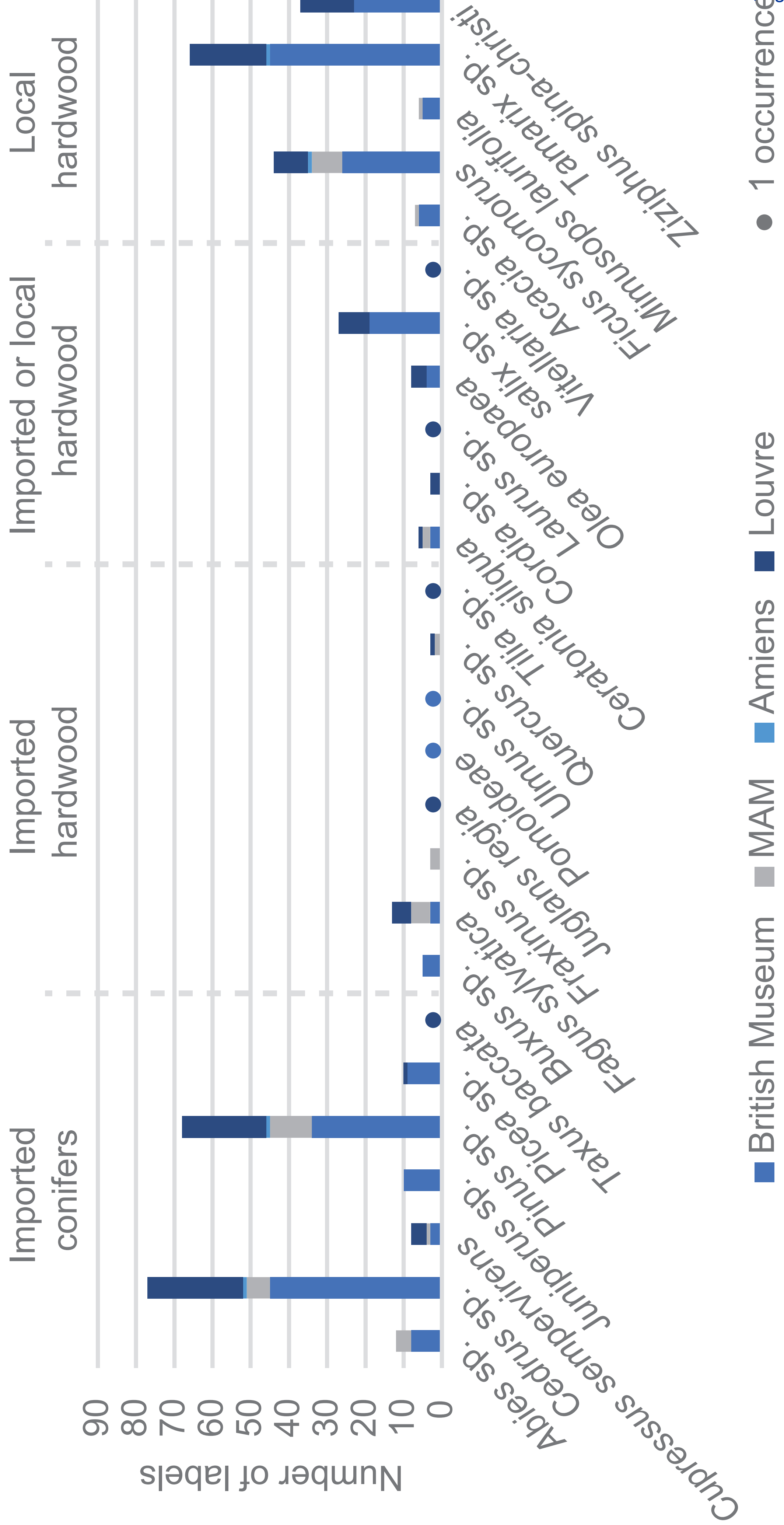

Figure 2

Click here to access/download;Figure;Figure2FB.eps

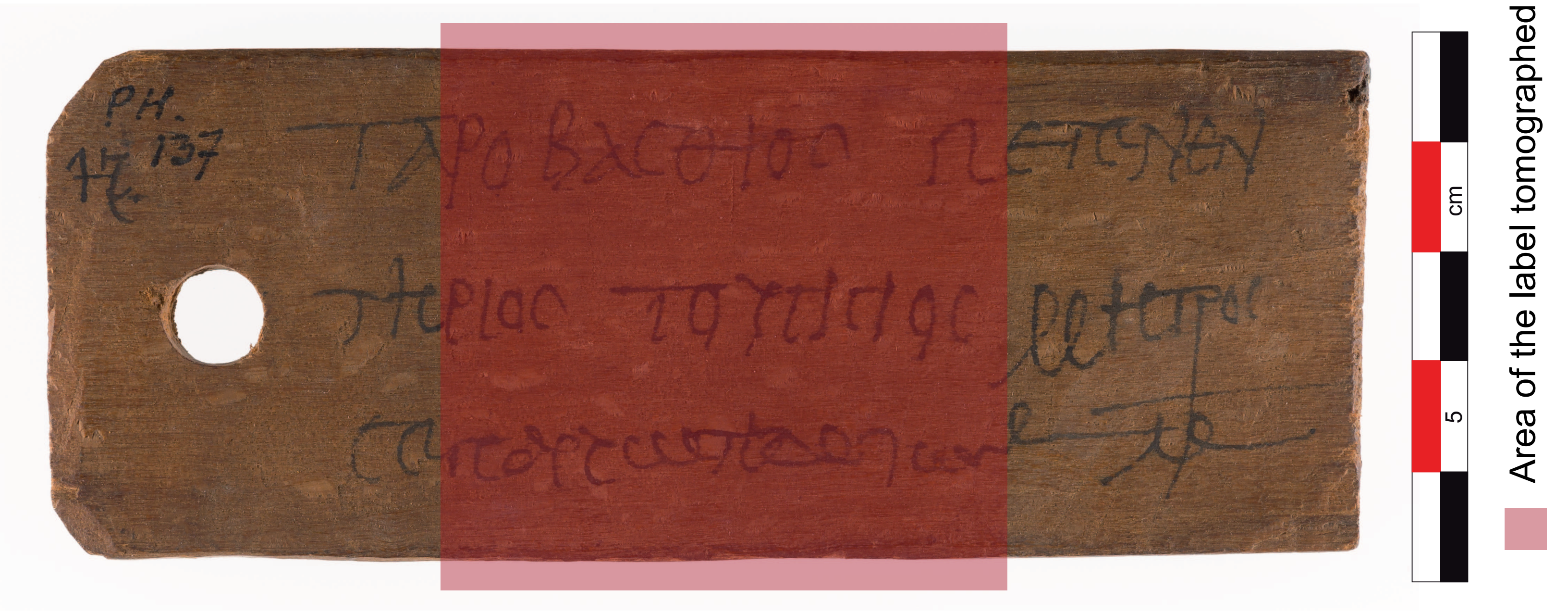

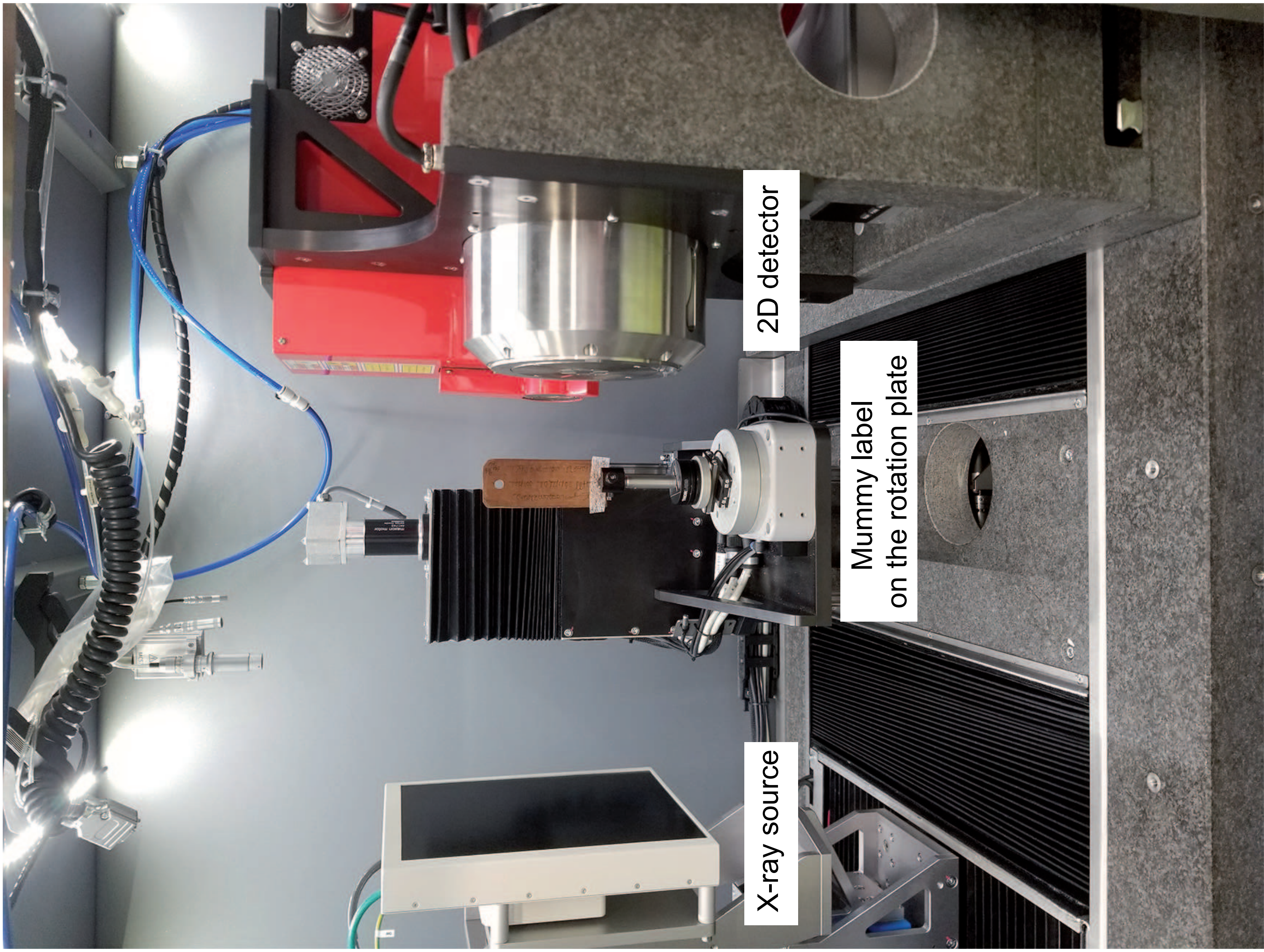

Click here to access/download;Figure;Figure4FB.eps

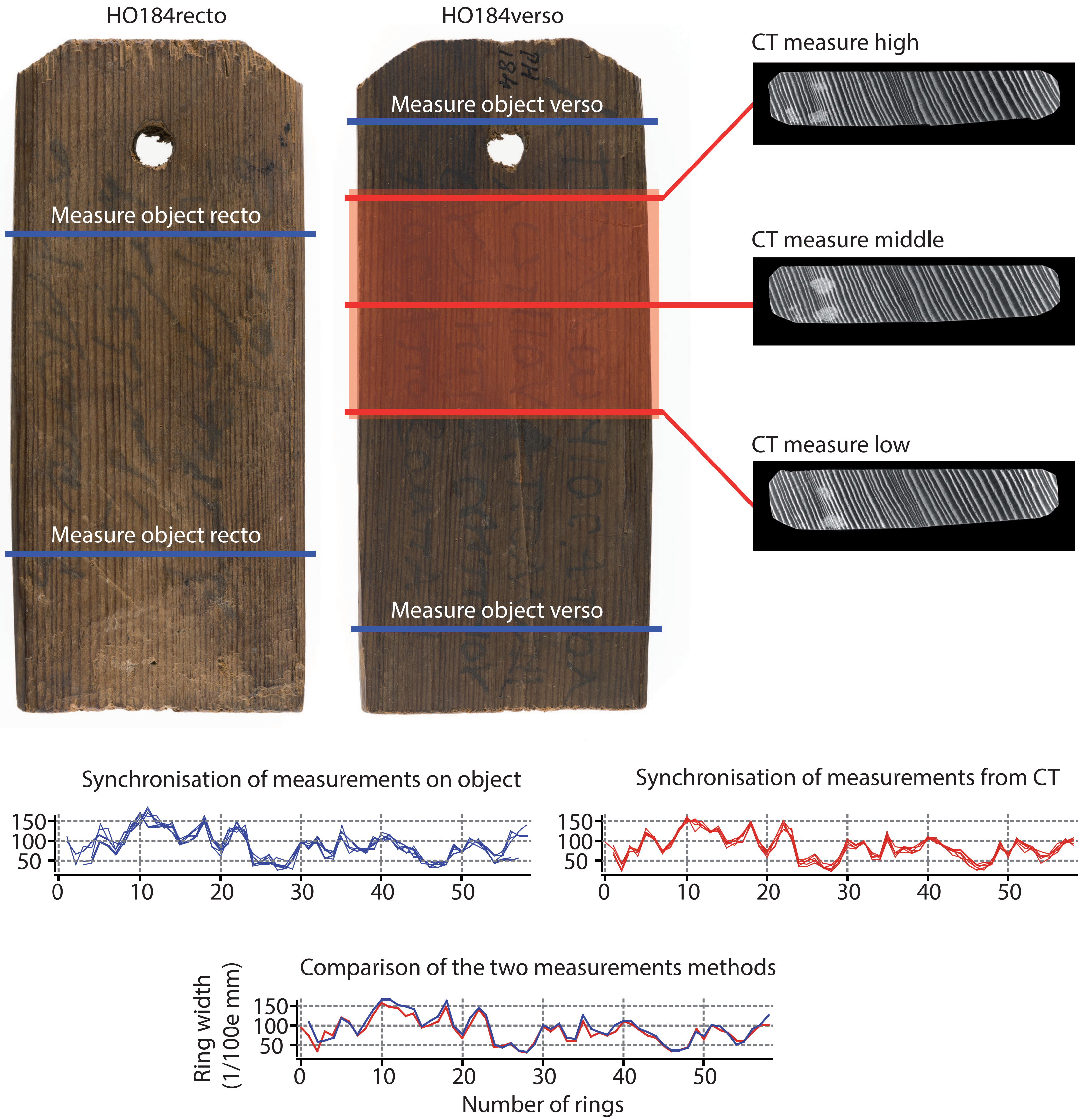

Supplement: Attachment — Submitted filename: PONE-D-23-11489 mummy labels with notes.pdf [file pone.0303695.s001.pdf]
