# Supplementary material for: The potential of X-ray computed tomography for xylological and dendrochronological analyses of Egyptian mummy labels
Source: PLoS One. 2024 Jun 28;19(6):e0303695. doi: 10.1371/journal.pone.0303695 (PMC11213358; doi:10.1371/journal.pone.0303695)
Supplement: Attachment — Submitted filename: rebuttal letter Plos One-v2.docx [file pone.0303695.s002.docx]

**Response to editor**

Thank you for your comments on our revised article. We have made stylistic changes in line with the templates. We have added the financial information requested on the site and removed this information from the acknowledgements and placed it in the dedicated section. As you indicated, I have also modified the cover letter to read: This article is part of an SNSF-funded post-doctoral project, project n°192176 "The Roman Egypt Laboratory:
Climat Change, Societal Transformations, and the Transition to Late Antiquity" (Project website: <https://ancientclimate.philhist.unibas.ch/en/project/> ; SNSF web page :
<https://www.snf.ch/fr>). The tomographic acquisitions of the 38 mummy labels were funded as part of SNSF project n°192176 "The Roman Egypt Laboratory. I also made it clear that the SNSF (the funders) did not influence the writing of the article and the results that followed from it.

It was our intention that the figures should be free of copyright. This was simply an oversight on our part when we first submitted them, as all the figures were produced for the article, except for figure 1 already used in a previous article, but is already in open access. They are therefore not subject to copyright. This error has now been corrected. We therefore authorise the publication of all the figures under the CC by 4.0 licence. In detail, I would like to point out that figure 1 was produced by S. R. Huebner, co-author of the article, and that figure 2 is a composition based on a photo of a label that I produced during tomography and on the detail of a label from the BNU collection (which is one of the photographs in the collection deposited on the zenodo server linked to the BNU website). Figure 3 is a figure that I created from the extraction of a tomographic image that I made from the dataset available on the zenodo server as part of the article. Figure 4 was produced from a montage of the front and back of the HO184 label (available on the server) and the extraction of views of the tomography dataset as well as the growth curves of my dendrochronological measurements. Figures 5, 6 and 7 are a montage of tomographic imaging that I created from the CT data set of several tags and images of the tool marks that I made myself. Figures 8, 9 and 10 consist of graphs from the anatomical, dendrological and dendrochronological observations that I made.

All the tomographic images of the 38 labels and the photographs of the latter taken by the BNU of Strasbourg, but in open access (public data) and a table summarising the tomograph settings for each label, as well as the views used to make the ring width measurements, have been archived as a compressed file and made available on a public repository (Zenodo). A text detailing the approach and protocol has been drafted and will be submitted to you. Data available on Zenodo will be made available upon acceptance of this article.

We do not understand that the reference "John J. Variation of wood anatomy in relation to environmental factors in two southern African broadleaves. Department of Pure and Applied Biology, imperial College, London, Unpublished PhD Thesis; 1990" is not acceptable, even though it is considered as such and is accessible on the imperial college of london website. But to be on the safe side, we have marked it as unpublished in the text and highlighted it in the bibliography, based on your comments. We have written a response to each of the reviewers. Corrections taken into account by the reviewers are highlighted in green. Some additional corrections have been made by the authors and are shown in purple in the commented text version.

**Response to Reviewers**

Reviewer 1

Thank you for your proofreading and the grammar and spelling corrections. Everything has been taken into account. We have specified how we selected the mummy labels according to their taxonomic diversity on the basis of initial observations under binoculars. This detail has been included in the "selection of tomographed labels" section.

Reviewer 2

We would like to thank you for your review and for your comments on our article. We have added a sentence to the introduction to point out the difficulty of establishing dendrochronological references for Egypt in general, and for the Roman period in particular, due to the practice of reusing wood. We have also modified tables 1 and 3, where the dimensions are in cm rather than mm. It was indeed a mistake on my part.

Reviewer 3

Thank you for your review and your many comments and remarks. You are right about the unrestricted availability of the data, and this is also a request from the SNSF. All the tomographic images have been deposited on a dedicated server so that they are accessible and the results reproducible. We have replaced "non-invasive" with "non-destructive" throughout the article. We have corrected and indicated the number of labels analysed in the summary and specified in the introduction between those taken into account in this study and the collection as a whole. Most of the French references have been replaced by English references, but some remain difficult to replace. I am thinking in particular of Catherine Lavier's work on archaeometric approaches to wooden collections from archaeology or museum collections, as this is not a case study but a global approach to possible approaches to these collections, or Béal Arnold's work, which is a mass of data and information on dendrochronological approaches to pirogues discovered in Europe, which also has no equivalent in English. We specified the Greco-Roman period as requested. Indeed, we had not given all the scan settings. All these details have been added to the method, along with confirmation that the settings were the same for all 38 labels. It is true that Figure 1 is the same as the article published in the IJWC, but PlosOne does not seem to have objected to including it in this new article. The reference to Bossema et al. 2021 has been added as requested. Finally, we have removed the acronym QWA in the conclusion.
